# Super-resolution microscopy reveals stochastic initiation of replication in *Drosophila* polytene chromosomes

**DOI:** 10.1007/s10577-021-09679-w

**Published:** 2022-02-28

**Authors:** Tatyana D. Kolesnikova, Galina V. Pokholkova, Viktoria V. Dovgan, Igor F. Zhimulev, Veit Schubert

**Affiliations:** 1 Institute of Molecular and Cellular Biology SB RAS Novosibirsk, Novosibirsk, Russia; 2 Novosibirsk State University, Novosibirsk, Russia; 3 Leibniz Institute of Plant Genetics and Crop Plant Research (IPK) Gatersleben, Seeland, Germany

**Keywords:** *Drosophila*, origin efficiency, polytene chromosome, replication initiation zone, replication timing, structured illumination microscopy

## Abstract

Studying the probability distribution of replication initiation along a chromosome is a huge challenge. *Drosophila* polytene chromosomes in combination with super-resolution microscopy provide a unique opportunity for analyzing the probabilistic nature of replication initiation at the ultrastructural level. Here, we developed a method for synchronizing S-phase induction among salivary gland cells. An analysis of the replication label distribution in the first minutes of S phase and in the following hours after the induction revealed the dynamics of replication initiation. Spatial super-resolution structured illumination microscopy allowed identifying multiple discrete replication signals and to investigate the behavior of replication signals in the first minutes of the S phase at the ultrastructural level. We identified replication initiation zones where initiation occurs stochastically. These zones differ significantly in the probability of replication initiation per time unit. There are zones in which initiation occurs on most strands of the polytene chromosome in a few minutes. In other zones, the initiation on all strands takes several hours. Compact bands are free of replication initiation events, and the replication runs from outer edges to the middle, where band shapes may alter.

**Supplementary Information:**

The online version contains supplementary material available at 10.1007/s10577-021-09679-w.

## Introduction

Eukaryotic replication origins are defined by origin recognition complex (ORC)-dependent loading of the Mcm2-7 helicase complex onto chromatin in the G1 phase (Bleichert et al. 2017). After loading onto chromatin, Mcm2-7 complexes are significantly redistributed across chromosomes and cover a substantial part of the genome with gaps within active transcription zones (Powell et al. 2015). Each potential replication initiation origin has certain efficiency and characteristic activation time. The origin activation time and efficiency are not related directly. Some later-activated origins are efficient, and others are not. Some origins are inefficient due to their proximity to earlier origins, while others are inefficient by themselves (Raghuraman et al. 2001; Weinreich et al. 2004). Origin efficiency in yeast varies widely and can reach 90% (Raghuraman et al. 2001; Heichinger et al. 2006). In metazoans, origins are much less efficient. Well-characterized origins fire in 5–20% of cells (Lebofsky et al. 2006; Hamlin et al. 2008). This low efficiency indicates that replication initiation events are probabilistic, and that their distribution may vary between different cell types and among subsequent cell cycles (Méchali 2010; Rhind et al. 2010; Herrick 2011; Wang et al. 2021). In recent years, several powerful approaches emerged that allowed genome-wide analysis of the probabilistic nature of replication initiation (Löb et al. 2016; Dileep and Gilbert 2018; Wang et al. 2021; Massey and Koren 2021). It is assumed that almost any site of the genome can initiate replication, but ORC assembly sites are considered the most efficient potential replication initiation sites (Wu and Nurse 2009; Borowiec and Schildkraut 2011; Gros et al. 2015; Powell et al. 2015; Miotto et al. 2016; Petryk et al. 2016). Usually, potential replication origins cluster to form so-called replication initiation zones. In humans, their median size is ~40 kbp (Tao et al. 2000; Dijkwel et al. 2002; Anglana et al. 2003; Hamlin et al. 2008; Borowiec and Schildkraut 2011; Lubelsky et al. 2011; Mesner et al. 2011; Bechhoefer and Rhind 2012; Besnard et al. 2012; Demczuk et al. 2012; Mesner et al. 2013; Petryk et al. 2016; Wang et al. 2021). When the first origin is activated within a cluster, neighboring origins become inactivated via interference with an extension of up to 100 kbp (Lebofsky et al. 2006).

**Fig. 1:**
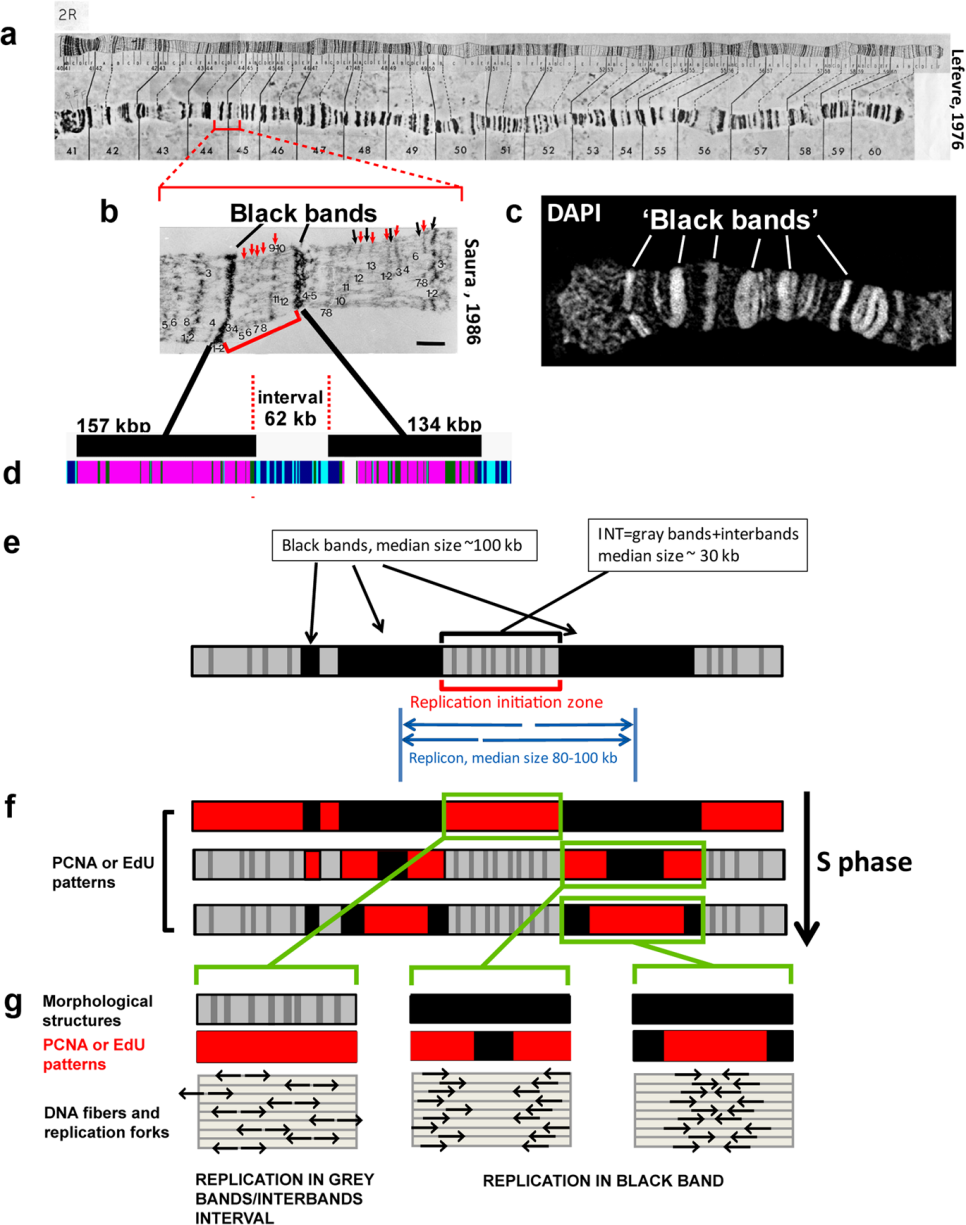
The spatiotemporal organization of replication in polytene chromosomes is closely related to the two main chromatin domain types present in polytene chromosomes: rb-bands and intervals (INTs) between them. **a** The classic photographic map of polytene chromosome 2R, on which dark “black” bands are clearly visible after aceto-orcein staining and phase contrast microscopy (Lefevre 1976). **b** A segment of chromosome 2R [an electron micrograph from the classic map of polytene chromosomes from Saura (1986); available in FlyBase: https://wiki.flybase.org/wiki/FlyBase:Maps]. Between the black bands are regions where decondensed gray bands (red arrows) and interbands (black arrows) alternate, together forming intervals (INTs). For two black bands and one INT between them, the sizes are indicated below according to genomic coordinates of these structures (Kolesnikova et al. 2018). **c** When stained with fluorescent dyes, black bands appear as the brightest regions on the polytene chromosomes (DAPI staining, 3D-SIM, this study). Gray band/interband structures in the INTs are faintly visible when stained with DAPI. **d** The Zhimulev et al. (2014) model of four states of chromatin (ruby, aquamarine, malachite, and lazurite) makes it possible to predict the localization of black bands in the genome based on the distribution of chromatin characteristics for black bands (chromatin called “ruby”: rb-bands). All interbands are associated with aquamarine chromatin, but not all aquamarine regions are interbands (Zhimulev et al. 2014; Boldyreva et al. 2017). The presented region corresponds to that in **b**. **e** A scheme of a polytene chromosome fragment bearing three black bands. Median lengths (in kilobase pairs) of black bands are shown, as are the intervals between them according to Kolesnikova et al. (2018) and the median sizes of replicons and replication initiation zones according to Lakhotia and Sinha (1983) and Schwaiger et al. (2009). **f** The scheme of replication patterns in polytene chromosomes and a model explaining the progression of these patterns [adapted from (Kolesnikova et al. 2018)]. Consecutive changes in PCNA-binding patterns (red) during the S phase. Three substages of the S phase are presented. From top to bottom: (i) early S phase, when INTs are labeled exclusively; (ii) middle S phase, when all rb-bands are labeled; and (iii) late S phase, when only the thickest rb-bands are labeled. **g** The scheme of replication fork locations and the respective PCNA/EdU patterns in the INT zone of alternating gray bands and interbands and in an rb-band (black). The arrows indicate the directions of replication fork progression

*Drosophila* salivary gland polytene chromosomes are composed of 2 × 1024 DNA strands representing both homologues. All strands are cohesively aligned in parallel. The visible band/interband pattern reflects the genome organization (Zykova et al. 2018). Interbands are the most decondensed regions with a specific protein composition conserved across tissues (Demakov et al. 2011, Vatolina et al. 2011, Zhimulev et al. 2014). According to their morphology, bands can be classified into gray and black. The most compact “black” bands, which look uniformly dense, even in sections imaged by electron microscopy (Fig. 1a, b), show a chromatin condensation ratio of 1:200 (Spierer and Spierer 1984; Kozlova et al. 1994; Vatolina et al. 2011; Zhimulev et al. 2014). After 4′,6-diamidino-2-phenylindole (DAPI) staining, these bands appear as the brightest ones (Fig. 1c) and correspond to clusters of tissue-specific genes. Besides, they contain silent chromatin and are almost devoid of ORC-binding sites (Zhimulev et al. 2014; Kolesnikova et al. 2018).

The 4-chromatin-states model of Zhimulev et al. (2014) was established based on the bioinformatic analysis of the distribution of interband-specific chromatin proteins in cell cultures (modENCODE Consortium et al. 2010). The *Drosophila* genome has been divided into four chromatin types formerly referred to as cyan, blue, green, and magenta (Zhimulev et al. 2014). Later, these four chromatin types were renamed as follows: aquamarine (cyan), lazurite (blue), malachite (green), and ruby (magenta). The genomic distribution of the four chromatin types is closely associated with the polytene chromosome morphology and allows to predict the genomic coordinates of polytene chromosome band boundaries with high accuracy because 1l experimentally characterized interbands coincide with aquamarine chromatin. Different combinations of the three other chromatin types correspond to different classes of bands (Zhimulev et al., 2014, Boldyreva et al. 2017, Kolesnikova et al. 2018, Demakova et al., 2020). The presence of ruby chromatin indicates a compact black band. Bands that do not contain ruby chromatin are referred to as gray bands (Kolesnikova et al. 2018).

The chromosomal regions between rb-bands are the alternation of thinner and less compact so-called gray bands and mainly decondensed interbands (Fig. 1b), which together form intervals (INTs) (Kolesnikova et al. 2018). INTs represent “open” chromatin and are enriched in genes expressed in different tissues. The promoters of these genes lie mainly within interbands, but the coding sequences within gray bands. According to cytological studies, the first replication marks during earliest S phase appear in INTs (Roy and Lakhotia 1979, 1981; Mishra and Lakhotia 1982; Kolesnikova et al. 2018). More than 90% of ORC2-binding sites are situated in aquamarine chromatin, primarily in INTs (Sher et al. 2012; Zhimulev et al. 2014; Kolesnikova et al. 2018). It seems that the ORC2-binding sites are markers of potential replication origins, although it is assumed that some replication initiation events may occur outside the ORC-binding sites in *Drosophila* and human (Eaton et al. 2011; Gros et al. 2015; Powell et al. 2015; Miotto et al. 2016; Petryk et al. 2016). Via immunostaining, the presence of the prereplication complex component DUP/Cdt1 was shown in all INTs analyzed along all polytene chromosomes (Belyaeva et al. 2012). Thus, all observations suggest that the vast majority of potential replication origins are located in INTs.

The visualization of replication in *Drosophila* polytene chromosomes by conventional wide-field (WF) microscopy indicates a well reproducible set of labeling patterns (Fig. 1f) (reviewed by Zhimulev 1999) reflecting the spatiotemporal organization of replication (Kolesnikova et al. 2018). At the beginning of the S phase, replication occurs in INTs. Then, continuous replication progresses until all chromosomes, except the middle parts of the thickest rb-bands, are labeled. Afterwards, an inversion of the pattern occurs: all rb-bands are marked but not the INTs. Finally, the rb-bands finish the replication. The more DNA an rb-band contains, the later is replication completed (Fig. 1f) (Zhimulev et al. 2003a; Kolesnikova et al. 2018). Intercalary heterochromatin bands and pericentromeric heterochromatin continue to replicate until the end of the S phase and remain under-replicated in salivary gland polytene chromosomes (Lakhotia 1974; Zhimulev et al. 1982, 2003b).

Proliferating cell nuclear antigen (PCNA) is a DNA clamp acting in eukaryotic cells and essential for replication (Moldovan et al. 2007). On the basis of PCNA immunostaining, Kolesnikova et al. (2018) analyzed the replication schedule of the entire chromosome arm 2R by WF microscopy. Additionally, an algorithm for mapping rb-bands to *Drosophila* genomic coordinates was developed (Kolesnikova et al. 2018). This helped to compare replication timing between polytene chromosomes of salivary glands and chromosomes from cultured diploid cell lines. Substantial similarities in the global replication patterns were observed between the two tissues. In that paper, we proposed the following model. In general, the spatiotemporal replication process is closely related to the genome organization into two types of domains corresponding well to the polytene chromosome structures: rb-bands and the INTs in between of them. INTs correspond to early replication initiation zones (Fig. 1e–g). The activation of replication in these zones occurs in time and space stochastically. On each individual chromatin strand, every interband belonging to an INT contains a potential replication origin. But only one of these potential origins (randomly chosen) activates replication in each cell cycle (Kolesnikova et al. 2018). This means that in polytene chromosomes inside one INT, different DNA strands initiate replication at different positions. We have indirect evidence for such a scenario: (1) In nuclei of cultured cells, INTs have an average replication profile corresponding to replication initiation zones in which replication is probabilistic in every replication cycle (Kolesnikova et al. 2018). (2) The characteristic replicon size is ~100 kbp, which is more than the average size of INTs (~30 kbp). This observation indicates that INTs cannot initiate replication on average more than once per cell cycle. (3) INTs have several interbands containing potential replication origins. That is, they correspond to the replication initiation zones, where (4) spatial replication asynchrony between DNA strands was observed in partially digested polytene chromosomes (Lakhotia and Sinha 1983).

To test this replication scenario directly in polytene chromosomes, here we used spatial super-resolution structured illumination microscopy (3D-SIM) as an efficient approach allowing multicolor detection of replication sites. In mammalian nuclei, the replication sites detected by 3D-SIM represent individual replicons (Schermelleh et al. 2008; Baddeley et al. 2010; Chagin et al. 2016).

It is challenging to analyze replication initiation in polytene chromosomes of wild-type nuclei because they replicate asynchronously. Therefore, we synchronized salivary glands by inducing S phases via ectopic expression of cyclin E by using a *D. melanogaster* line carrying the *hsp70-CycE* transgene (Knoblich et al. 1994; Duronio and O’Farrell 1995; Su and O’Farrell 1998).

By means of induced-S-phase analysis, 5-ethynyl-2′-deoxyuridine (EdU) replication detection, and super-resolution analysis, in the present study, we confirm the hypothesis of stochastic replication initiation in salivary glands at the ultrastructural level. We demonstrate that the multifilament nature of polytene chromosomes provides unique opportunities for visualizing stochastic processes within chromatin.

## Materials and methods

### Flies

Flies were reared on enriched semolina (36 g/l) medium with raisins at 18 °C. The Oregon R (Bloomington Stock Center) stock served as a control. The *hsp70-CycE* line was kindly provided by C. Lehner (University of Zurich, Switzerland) and by P. O’Farrell (UCSF, USA). Additionally, we constructed the *hsp70-CycE; SuUR* line. In contrast to wild-type chromosomes, intercalary and some regions of pericentromeric heterochromatin remain not under-replicated in *SuUR* mutants (Belyaeva et al. 1998).

### EdU incorporation and detection

Actively moving wild-type third instar larvae were employed for replication analysis because they have a higher percentage of nuclei in S-phase stages. To analyze replication in induced S phase, larvae shortly before pupation were used, when the last larval S phase is already finished (Zhimulev et al. 2003a; Kolesnikova et al. 2013) and many nuclei enter the additional induced S phase. This approach provided an additional round of replication.

Salivary glands were dissected and stored in 1 × PBS (137 mM NaCl, 3 mM KCl, 8 mM NaH_2_PO_4_, 2 mM KH_2_PO_4_). EdU incorporation was carried out in a 4 μM EdU solution in 1 × PBS for 10 min. Salivary glands were placed into 1 × PBS supplemented with 0.1% of Tween 20 (1 × PBST) for 2 min incubation, transferred to a formaldehyde-based fixative (2% NP40 and 2% formaldehyde in 1 × PBS) for 2 min incubation, incubated in an acetic acid–formaldehyde mixture (45% acetic acid, 3.2% formaldehyde) for 1.5 min, and squashed in 45% acetic acid. The squashes were snap-frozen in liquid nitrogen, and the coverslips were removed. The slides were stored in 70% ethanol at −20 °C. For EdU detection, the Click-iT™ EdU Alexa Fluor™ 555 Imaging Kit (Thermo Fisher Scientific) was used. The slides were washed first in 1 × PBS for 20 min, incubated in PBST with 0.1 BSA for 30 min, and then treated with a reaction cocktail at room temperature for 30 min (40 μl/slide). After three 5-min washes in 1 × PBST, the slides were air-dried by means of a rubber syringe, and the squashes were mounted in the VectaShield (Vector Laboratories) medium containing 1.5 µg/ml DAPI.

### Combination of EdU detection and FISH

To obtain probes for FISH, genomic DNA was amplified by PCR using the following primers: *lola 5’* (aaagatggtctcggcttgtgt and gtcgctccgctcgttaaattc); *lola 3’* (tcaagagagcgggtgagtttc and ccacagtgaagatcagccagt), and *Sgs3* (gcatcacgcggtattgaattcc and cttcttgcctgaatcacacgc). DNA probes were labeled with either Tamra-5-dUTP or FLu-12-dUTP (Biosan, Russia) in a random-primed polymerase reaction using the Klenow fragment (SibEnzyme, Russia).

Salivary glands of third instar *hsp70-CycE; SuUR* larvae were dissected in 1 × PBS solution 70–90 min after 30 min heat shock, incubated with EdU, and placed in a fixative (96% ethanol: acetic acid, 3:1) for 4–5 h. Slides were squashed in 45% acetic acid, frozen in liquid nitrogen, the coverslips were removed, and the slides were stored in 70% ethanol at −20 °C.

Slides were treated with RNAse A. For this, slides were washed in 2 × SSC (0.3 M NaCl, 0.03 M sodium citrate) 3-times for 5 min and incubated with RNAse A (100 μg/ml in 2 × SSC, 30 μl/slide) for 1 h at 37 °C and washed in 2 × SSC for 5 min. Then, slides were incubated in 2 × SSC for 1 h at 60 °C, denatured in 0.07 M NaOH, passed through a series of ethanol (70, 90, 96%) for 5 min, and air-dried. Afterwards, to detect EdU, slides were treated as described above. After the final washing, the hybridization mixture was added to the slides. To prepare this mixture, 4 μl of a labeled probe, 5 μl of water, and 1 μl of a calf thymus DNA solution were mixed in a tube and heated at 95 °C for 5 min, then cooled and centrifuged. To the same tube, 20 μl of warm hybridization solution (50% formamide, 2 × SSC, 10% dextran sulfate) was added, and then 30 μl of this mixture applied per slide. The hybridization was performed for 1.5–2 days in a humid box at 37°C. The unbound probe was removed by three 15-min washes in 0.2 × SSC with a gradual increase in temperature (42–60 °C). Slides were mounted in VectaShield (Vector Laboratories) containing 1.5 μg/ml DAPI.

### Indirect immunostaining

For polytene chromosome immunostaining, salivary glands (genotypes are specified in the text) were dissected in 1 × PBS supplemented with 0.1% of Tween 20. The glands were then transferred into a formaldehyde-based fixative (0.1 M NaCl, 2 mM KCl, 10 mM NaH_2_PO_4_, 2% of NP40, 2% of formaldehyde) for 1 min incubation. Next, the salivary glands were placed in an acetic acid–formaldehyde mix (45% acetic acid, 3.2% formaldehyde) for 1 min incubation and squashed in 45% acetic acid.

The squashes were snap-frozen in liquid nitrogen and the coverslips removed. Then, the slides were incubated in 70% ethanol for 5 min twice and stored in 70% ethanol at −20°C. The slides were washed three times in 1 × PBST (137 mM NaCl, 3 mM KCl, 8 mM NaH_2_PO_4_, and 2 mM KH_2_PO_4_; 0.1% of Triton X-100 or Tween 20) for 5 min. Mouse anti-PCNA (PC10, Abcam, ab29, 1:500) or mouse anti-RNAPIIser2ph (Abcam, ab5095, 1:400) primary antibodies were added into a blocking solution (0.1% BSA in 1 × PBST) and incubated on the slides in a humidified chamber for 2 h at room temperature. The squashes were washed 3 times for 5 min in 1 × PBST, and secondary antibodies (Alexa Fluor 488– or Alexa Fluor 568–conjugated goat anti-mouse IgG antibodies; 1:500; Thermo Fisher Scientific) diluted in the blocking solution were applied. After 1 h incubation followed by washing with 1 × PBST 3-times for 5 min each, slides were mounted in VectaShield (Vector Laboratories) containing 1.5 µg/ml DAPI.

For combined immunostaining and EdU labeling, the glands were first labeled with EdU as described above, then the preparations were first stained with antibodies, and afterwards, EdU was detected as described above.

### Super-resolution microscopy

To analyze the ultrastructural organization of replication beyond the classic Abbe–Rayleigh limit of ~250 nm, 3D-SIM was performed to achieve a lateral resolution of ~140 nm (super-resolution, attained with a 561 nm laser). We used an Elyra PS.1 microscope system equipped with a Plan-Apochromat 63×/1.4 oil objective and the ZENBlack software (Carl Zeiss GmbH). Image stacks were captured separately for each fluorochrome by means of 405 nm (DAPI), 488 nm (Alexa Fluor 488), and 561 nm (Alexa Fluor 555) laser lines for excitation with appropriate emission filters (Weisshart et al. 2016). Zoom-in sections are presented as single slices to detect the subnuclear chromatin structures at the super-resolution level.

Movies were prepared based on 3D-SIM image stacks using the Imaris 9.7 (Bitplane) software.

To investigate the spatial chromatin ultrastructure, 3D-SIM was performed on ~30 optical sections from ~3 μm thick chromosomes. By significantly increasing the resolution, 3D-SIM showed clearly more structural details in comparison with WF microscopy, as demonstrated by imaging of different replication patterns during S-phase progression (Supplementary Fig. 1a, b). Orthogonal projections indicated that the polytene chromosomes preserve their cylindrical shape after the fixation applied. The EdU signals were conspicuous within the chromosomes, both in decondensed regions (the pattern characteristic for early replication) and inside compact bands (the pattern characteristic for late S phase; Supplementary Fig. 1c). Thus, to analyze the replication patterns in more detail, we employed exclusively 3D-SIM in this work.

We analyzed 75 nuclei on 10 slides by 3D-SIM (Table 1).

**Table 1:** Numbers of polytene chromosome nuclei analyzed by 3D-SIM

Genotype	No. of preparations	No. of nuclei stained by anti-PCNA	No. of nuclei stained via EdU incorporation
Wild type	2	8	17
*hsp70-CycE; SuUR*, without heat shock	3	6	6
*hsp70-CycE; SuUR*, 90 min after heat shock	3	3	21
*hsp70-CycE; SuUR*, 120 min after heat shock	1	-	5
*hsp70-CycE; SuUR*, 180 min after heat shock	1	-	8

### S-phase induction

To induce S phases in salivary glands, cyclin E was expressed ectopically (Knoblich et al. 1994; Duronio and O’Farrell 1995; Su and O’Farrell 1998) in *hsp70-CycE; SuUR* third instar larvae. To obtain more simultaneously induced S phases in salivary glands, the *hsp70-CycE; SuUR* larvae were heat shocked at the end of the 3rd instar, a period with a minimum of S-phase cells (Kolesnikova et al. 2013). First, we followed previous protocols (Duronio and O’Farrell 1995; Su and O’Farrell 1998) and applied a heat shock at 37 °C for 30 min. Ca. 30 larvae were analyzed by incorporating EdU into the salivary glands isolated 50 min–6 h after the heat shock. After 70 min, we found in some preparations a slight enrichment of nuclei at very early S phase. In glands prepared 2–3 h after heat shock, the proportion of labeled nuclei reached 100%. However, due to the non-simultaneous entry of the cells into S phase, no high synchronization could be achieved. It was published that after the 37 °C heat shock, the cells do not recover synchronously after the transcription block, but at 35 °C, transcription becomes restored at the end of the heat shock (Kutskova and Mamon 1995; Gong and Golic 2006). After the 35 °C (for 30 min) heat shock, all analyzed salivary glands isolated 70 min after heat shock showed stable groups of nuclei at very early S phase. Ninety minutes after heat shock, all nuclei were labeled, what never occurred in glands without heat shock. In total, more than 70 slides 70–90 min after 35°C heat shock were analyzed by WF microscopy.

Besides, we analyzed by WF microscopy slides after 10 min EdU incorporation into the salivary glands isolated 2, 3, 4, 5, 6, 7, and 8 h after heat shock (about 10 slides per moment) from *hsp70-CycE; SuUR* larvae. Up to 3 h after heat shock, the induced S-phase dynamics could be observed. The nuclei with induced S phase were distinguished from the others by the predominance of them at the same early S-phase stages (early and early-middle). When analyzing glands isolated 3 or more hours after heat shock, we found an enrichment of stages where all compact bands of the polytene chromosomes were stained (middle S phase). But we could not identify nuclei related to induced and non-induced S phase. In addition, the high percentage of nuclei at middle S phase in larvae 7–8 h after heat shock suggested that the S phase is inhibited, as this pattern is similar to the pattern after hydroxyurea treatment (Kolesnikova et al. 2013). Thus, we showed that our approach is suitable for studying early S-phase stages, but not for analyzing the S-phase timing more than 2–3 h after S-phase initiation.

To investigate the reproducibility of early replication patterns after S-phase induction, the EdU signal distributions were analyzed in 24 nuclei from three preparations of *hsp70-CycE; SuUR* larvae (90 min after heat shock) compared to wild-type. We compared several selected regions on three preparations (Supplementary Figs. 2, 3a, b, and 4a). Supplementary Fig. 4a illustrates very early replication in the 10A-11A region of chromosome X. On slides 1 and 2, replication was detected by the EdU incorporation assay, and on slide 4, by PCNA immunostaining. A region of early replication lies between late-replicating rb-bands 10B and 11A (Belyaeva et al. 2012). The chromatin morphology (after DAPI labeling) was well reproducible, but small differences in the stretching degree of the chromosomes due to squashing were noticeable. EdU and PCNA signals were similar. Consequently, we conclude that for early replication detection, both markers are suitable for 3D-SIM analysis. Although the signals were reproducible overall, there were some differences between and within the slides. The intraslide differences were primarily related to incomplete synchronization of the cells entry into the S phase. On different slides, there were various chromosome stretching degrees and differences in the appearance of all signals. The scheme in Supplementary Fig. 2d presents a hypothetical explanation of the effect of chromosome stretching on the visible replication pattern.

The analysis of the dynamics of replication timing showed that it is most appropriate to use nuclei from the same preparation, because then, the chromosome stretching is comparatively uniform and the stages of development are identical.

### Genomic mapping of polytene chromosome bands: a comparison of cytological and molecular data

To map cytological regions to genomic coordinates, we applied the approach of Kolesnikova et al. (2018). All the genomic coordinates used in this work are available in the *D. melanogaster* Release 5 assembly. The UCSC Genome Browser (Meyer et al. 2013) was employed for data visualization. Genomic positions of rb-bands were identified using the coordinates of four chromatin types established by Zhimulev et al. (2014). The CHRIZ protein distribution in cell cultures (modENCODE_275, modENCODE_277, modENCODE_278, and modENCODE_276), SUUR (Maksimov et al. 2014) and H3K27me3 (Posukh et al. 2017) protein distributions in salivary glands, and data from in situ hybridization (available from the FlyBase database) were used for more detailed mapping of band edges. Additionally, we utilized photographs (kindly provided by Todd Laverty) from in situ hybridization assays of P inserts from the BDGP project (Spradling et al. 1999). Insert or target gene names served as queries for retrieving the genomic coordinates of the inserts from FlyBase. Hi-C data for salivary glands (Eagen et al. 2015) were analyzed using accession GSE72512 in Gene Expression Omnibus.

ORC2 distribution data were taken from Sher et al. (2012) (GSE31899). Data on the distribution of early origins of replication in Kc cells were borrowed from MacAlpine et al. (2010) (GSE17285).

### Computer simulation of replication

The replication simulation for a polytene chromosome fragment was carried out by a computer program written in the Delphi Pascal language (Supplementary Text 1).

We regarded a polytene chromosome as a number (*N*) of similar threads. In each thread, replication was assumed to be initiated stochastically. Given that the threads are exact copies of each other and are precisely aligned, we assumed that in all the threads, positions of potential replication initiation sites and the probability of replication initiation are identical. Therefore, the distribution of replication initiation sites on *N* DNA strands should reflect the distribution of initiation probabilities for one strand. We examined *N* = 1024 threads, which is the number of DNA strands in one homolog of a polytene chromosome (late third instar larvae).

The rate of movement of replication forks along a DNA strand was considered constant. Accordingly, replication durations along the chromosome axis in interbands, gray and rb-bands depended on the DNA content. This assumption is a simplification because the actual speed in late-replicating bands is ~10 times lower as that was shown for *D. nasuta* polytene chromosomes (Lakhotia and Sinha 1983). Our evaluation of the replication fork movement speed was based on the following assumptions. In *D. melanogaster*, the mean replication fork rate is ~0.24 kbp/min for late replicons (Kolesnikova et al. 2009). If we suppose that during the very early S phase, the speed is ten times higher, then we get ~2 kbp/min.

Visible thickness of the bands and interbands along the chromosome axis depends on the stretching degree of the chromatin. DNA packing ratios in interbands are 3–15, in gray bands 54–63, and in black bands >200 (Spierer and Spierer 1984; Kozlova et al. 1994; Vatolina et al. 2011; Zhimulev et al. 2014). When modeling, we depicted gray bands and interbands with the same thickness for simplification. According to the four-color chromatin model, all interbands correspond to aquamarine chromatin, with average and median sizes of 2.7 and 1.8 kbp, respectively (Zhimulev et al. 2014; Boldyreva et al. 2017), consistently with the Beermann (1972) estimate of 2 kbp DNA per interband. Thus, we chose an interband size of 2 kbp.

The thickness of polytene chromosome structures cytologically revealed as gray bands varies considerably and ranges from 2 kbp (the minimum band visible by electron microscopy in semithin sections) to tens of kilobase pairs (Vatolina et al. 2011; Demakova et al. 2020; Khoroshko et al. 2020). In the present work, we estimated the size of the 21C6 gray band to be ~60 kbp. Thick and thin gray bands were found to differ significantly in morphology. The thinnest ones are detectable only by electron microscopy and on the very best preparations, while the bands containing more chromatin are sometimes very similar to black bands. Therefore, we introduced two types of gray bands into the model. The first type corresponds to 5 kbp of DNA and is only 2.5-fold more compact than the interbands, while the second type is 20 kbp long and 10-times compacter. Accordingly, we simulated an INT of 6 × 2 kbp + 4 × 5 kbp + 20 kbp = 52 kbp. This is close to the average size and 1.5-fold larger than the median size of INTs. The INTs sizes vary from 0.5 to 300 kbp on chromosome 2R (Kolesnikova et al. 2018).

## Results

### S-phase induction by cyclin E is reliable to analyze the early replication dynamics in polytene chromosomes

**Fig. 2:**
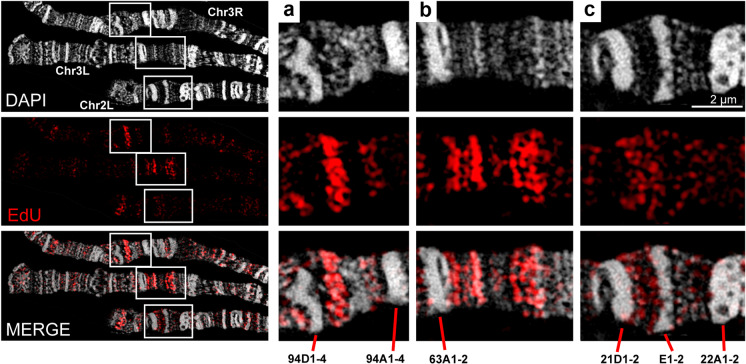
Replication in wild-type polytene chromosomes is initiated in band-shaped (**a**, **b**) and local diffuse patterns (**c**) in the very early S phase within INTs. The enlarged regions in **a**–**c** are indicated by rectangles in the overview image (left)

Previously, it was demonstrated that in actively moving wild-type larvae, when grown at 18 °C, early S-phase stages in the salivary glands occur with a frequency of up to 50% (Kolesnikova et al. 2013). A small proportion of the nuclei is at a very early stage when replication starts only in several decondensed regions representing INTs between compact bands as well as in several puffs. Local band-shaped and more diffuse patterns were found (Fig. 2). The INT between bands 94D1-2 and 94A1-4 of chromosome 3R is an example of a bright local signal pattern. The EdU signals lie in the loose chromatin that does not form a clear band, and the signals are concentrated in a small region of the INT (Fig. 2a). A similar situation was observed in the region of the thin gray bands to the right of the compact band 63A1-2 (Fig. 2b). Here, the signals were seen as two band pairs. Figure 2c depicts two intervals between rb-bands (INTs 21D1-2/21E1-2 and 21E1-2/22A1-2) of chromosome 2L. Spatial image reconstruction (Supplementary Movie 1) showed that the signals are evenly distributed within the entire INTs. This pattern is consistent with our assumption that INTs match replication initiation zones emerging in different strands and origins.

It is challenging to analyze replication initiation in polytene chromosomes of wild-type larvae because the nuclei in a salivary gland do not replicate synchronously. Therefore, all conclusions about S-phase progression were based on circumstantial evidence. To obtain more information about the initiation of replication in polytene chromosomes and to reveal temporal dynamics of the early S phase, we synchronized salivary gland cells by inducing the S phase via ectopic expression of cyclin E. For this purpose, we used a *D. melanogaster* line carrying the *hsp70-CycE* transgene (Knoblich et al. 1994; Duronio and O’Farrell 1995; Su and O’Farrell 1998). Additionally, to obtain a better chromosome morphology, we introduced the *SuUR* mutation into the line. The *SuUR* mutation affects the progression of replication forks in silent chromatin regions, primarily in the regions of pericentromeric and intercalary heterochromatin. This leads to suppression of under-replication but does not affect replication initiation (Sher et al. 2012). Previously, we reported that this mutation, which influences the late-replication pattern, does not affect the very beginning of the S phase (Kolesnikova et al. 2013). Late third instar *hsp70-CycE; SuUR* larvae and 0 h prepupae were subjected to a 35°C heat shock (see “Materials and Methods” for details). Then, we kept the larvae at room temperature and isolated the salivary glands after different time intervals, incubated them with 4 μM EdU for 7–10 min and fixed them immediately. Seventy minutes after the heat shock, the first induced S phases became visible. After another 20 min, the proportion of labeled nuclei reached 100%. This result suggests that the cells enter the S phase with a temporal shift of ~20 min. The induction at this stage of development allowed to obtain a high proportion of nuclei at very early S-phase stages, which is normally very low (Zhimulev et al. 2003a; Kolesnikova et al. 2013). Besides, we obtained an additional round of polytenization, leading to larger and better-structured polytene chromosomes (Supplementary Fig. 3a, b).

In diploid cells, S-phase activation by cyclin E impairs the distribution of replication initiation sites. This effect may cause conflicts between replication and transcription and induce carcinogenesis (Teixeira and Reed 2017; Macheret and Halazonetis 2018). Therefore, we checked whether S-phase induction via cyclin E overexpression altered the early replication patterns in polytene chromosomes. We compared the early patterns of normal and induced S phases. No differences were detectable as exemplified by region 56A-57B of chromosome 2R in Supplementary Fig. 3c. The pattern of middle replication was also similar to the control, as visible in the specimen prepared 180 min after S-phase induction. Our approach did not allow analyzing the induced S phases at later stages, but the patterns of late replication observed in nuclei that were in S phase before heat shock were normal. We conclude that CycE overexpression does not led to qualitative changes in replication patterns in preparations made 1–3 h after heat shock induction.

In mammalian cells, forced expression of cyclin E abridges G1 phase, resulting in premature S-phase entry with prereplication complexes still present at the 3′-end of long genes. Normally, prereplication complexes become removed by active transcription during G1 (Macheret and Halazonetis 2018). To check whether there is an ectopic initiation of replication within highly expressed long genes, we analyzed the localization of loci of highly efficient early initiation simultaneously with the detection of active transcription. Supplementary Fig. 5 shows the earliest replication pattern of a nucleus at a stage when less than 30 bright EdU signals can be clearly identified, but the rest is still not yet activated. Some of these signals are close to bright transcriptional signals, others not. Many transcriptionally active regions do not show early, highly efficient replication signals. We chose locus 47B for a detailed analysis. Here, we found early replication and active transcription signals close together. The rb-bands 47A1-2 and 47B4-5 were mapped on the genomic map earlier (Kolesnikova et al. 2018). According to ModEncode project data in salivary glands, the most actively transcribed gene within this locus is the long (~50 kb) gene *lola*. Multiple ORC2 sites are located at the 3′ and 5′ ends of this gene (Sher et al. 2012). Besides, the 5′ region has the highest peak of “early origins” in the Kc cell culture. We assumed that the upstream intergenic region of the lola gene induces the bright replication signal, while the lola gene is responsible for the active transcription. To prove this, we performed FISH with probes corresponding to the 5′ and 3′ gene regions. The probes localized at both sides of the gray band, i.e., the band contains a transcribed part of the *lola* gene. Simultaneous FISH and RNAPIIser2ph detection, and FISH and EdU incorporation demonstrated that the bright signals of early replication lie right to the transcription signals, partially colocalize with the 5′ probe, but are clearly separated from the 3′ probe. Thus, we conclude that the replication initiation is confined to the non-coding region near to the active gene promoter. Due to the small number of early signals, we consider them as specific. We conclude that S-phase induction is a reliable technique to analyze early replication dynamics in polytene chromosomes.

### At the very beginning of the S phase, replication is initiated differently in various INTs but similarly in salivary glands and diploid cells

**Fig. 3:**
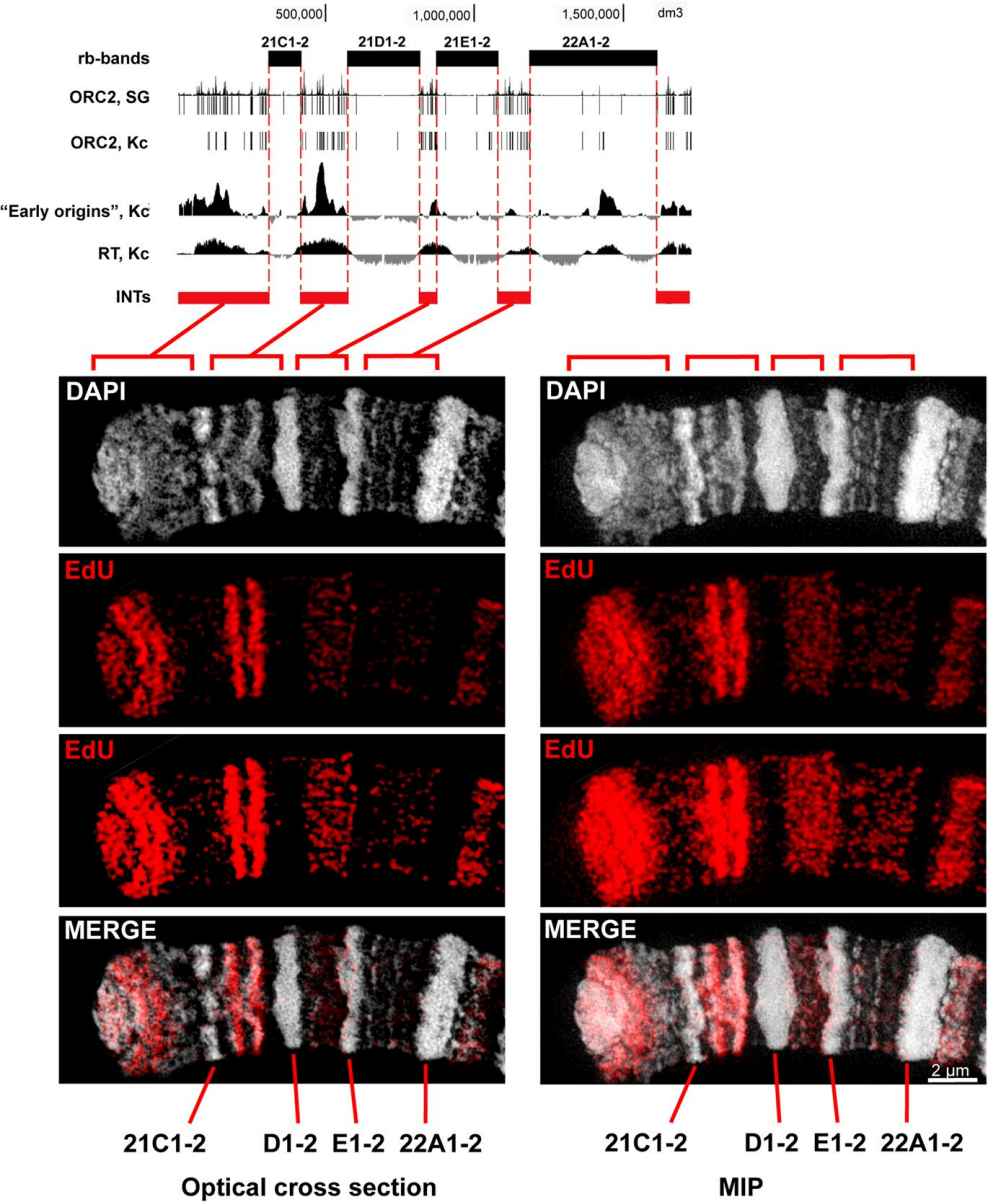
Early replication in the distal part of chromosome 2L. The region contains rb-bands (21C1-2, 21D1-2, 21E1-2, and 22A1-2) and INTs, as pointed out in the scheme by black and red rectangles, respectively. The replication parameters within the region are based on published data. The distribution of ORC2 proteins in salivary glands (SGs) and the Kc cell line is based on data of Sher et al. (2012). The distribution of newly synthesized DNA in the presence of hydroxyurea (“Early origins”) in S2 cells was published by MacAlpine et al. (2010). RT, Kc: replication timing profiles in the Kc cell line reported by Schwaiger et al. (2009). The corresponding optical cross-section and maximum intensity projection (MIP) of a 3D-SIM image stack below the scheme illustrate the very early replication pattern via EdU incorporation. The two variants of representation, differing in brightness, are presented to show all details of weak and bright signals. The red brackets indicate the sections of the chromosome matching to the INTs. The specimen was prepared from *Hsp70-CycE; SuUR* larvae, 90 min after heat shock

While analyzing very early replication patterns, we found that the signals are distributed unevenly in different chromosomal regions. To demonstrate this observation, we chose the distal region of chromosome 2L as an example (Fig. 3, Supplementary Movie 2).

This polytene chromosome region has not yet been mapped to genomic coordinates. Therefore, we applied the algorithm of Kolesnikova et al. (2018) to determine the position of big bands. Four large bands enriched with “ruby” chromatin (Zhimulev et al. 2014) were predicted. Relative positions and sizes of these bands exactly match bands 21C1-2, D1-2, E1-2, and 22A1-2 (Supplementary Fig. 6). Consequently, these four bands can be assigned to rb-bands. Between rb-bands 21C1-2 and D1-2, two thinner and less compact bands (21C4 and 21C6) are evident.

The analysis of the EdU distribution corresponding to the very early S phase in the 2L chromosome region presented in Fig. 3 shows mostly the absence of EdU signals in rb-bands. All INTs contain EdU signatures, but intensity levels and distributions are significantly different (Supplementary Movie 2).

The INT to the left of 21C1-2 is characterized by a bright signal pair (in Fig. 3 on the left) with relatively bright diffuse signals toward the telomere and rare diffuse signals in the rest of the chromosome region. Obviously, in this INT, the initiation does not occur uniformly. There is a zone where one or more highly efficient origins are localized.

In the INT 21C1-2/21D1-2, a very bright signal pair, confined to bands 21C4 and 21C6, stands out (Fig. 3, Supplementary Movie 3).

In INTs 21D1-2/21E1-2 and INT 21E1-2/22A1-2, the EdU signals are homogeneously distributed within the whole INT volumes (Fig. 3; Supplementary Movies 2, 4, and 5), while in INT 21D1-2/21E1-2, the signals seem to be more clustered and are brighter and larger, possibly indicating a replicon grouping. In INT 21E1-2/22A1-2 of the Fig. 3, the signals appear as 102 dots. This number means that at this time, less than 10% of the DNA strands initiated replication, suggesting that the initiation process proceeds gradually, which is consistent with data from autoradiographic analysis of replication (Lakhotia and Sinha 1983). In INT 21E1-2/22A1-2, thin bands are well discernible, which indicates that the DNA is precisely aligned along the chromosome axis. In contrast, the EdU signals appear to be distributed homogeneously. This distribution is in good agreement with the hypothesis that INTs act as replication initiation zones where any interband can initiate replication.

Both INTs of chromosome 2L showing the bright band-shaped EdU signals correspond to peaks of newly synthesized DNA in the presence of hydroxyurea presumably relevant to “early origins” in S2 cells. These signals probably represent early and efficient origins acting in both salivary glands (our data) and S2 cells [according to MacAlpine et al. (2010)]. The other three INTs also have peaks of “early origins,” but they are significantly lower than the peak in the INTs between 21С1-2 and 21D1-2. The concentration of EdU signals in the INTs correlates with the peak heights in S2 cells (Fig. 3). A similar correlation between the highest peak of “early origins” and the strongest signal of early replication in salivary glands is present in region 47A-B (Supplementary Fig. 5).

In short, it can be concluded that different INTs initiate replication differently and that the origin efficiency is similar between salivary glands and diploid cells.

### Early replication is highly dynamic

**Fig. 4:**
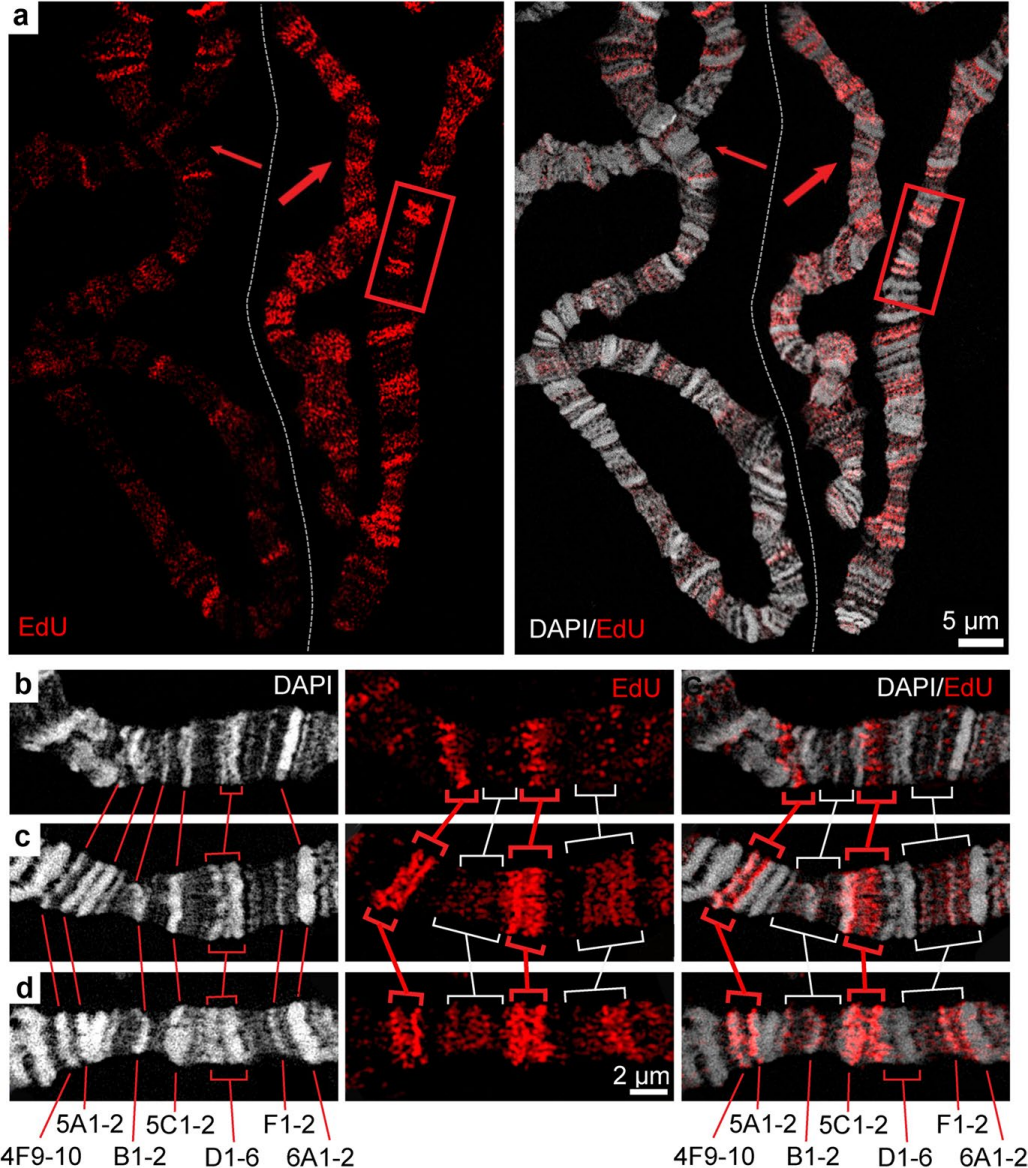
S-phase induction helps to study replication dynamics within the first minutes after induction. **a** Chromosomes from two nuclei in different early S-phase stages (two arrows). The left-hand chromosome shows the earliest pattern where rare bright signals lie in puffs and loose bands. The right-hand nucleus features a later pattern. **b–d** The temporal dynamics of early replication with an example of X chromosome region 4F-6A from the same preparation as in **a**; **d** is the framed region in **a**. The specimen was prepared from *Hsp70-CycE; SuUR* larvae, 90 min after heat shock. Given that the first nuclei enter the S phase 70 min after the shock, the temporal distance between the patterns is ~20 min

EdU signal intensity varies significantly among the nuclei prepared 90 min after heat shock (Fig. 4). Considering that the S-phase induction is shifted in different nuclei, a comparison of the patterns allows to make a conclusion about the temporal dynamics of replication during the first 20 min of the S phase. In Fig. 4a, two chromosomes of neighboring nuclei are presented. They differ in total EdU signal intensity. The left nucleus has an EdU pattern typical for the specimens prepared 70 min after heat shock (data not shown). The overall signal intensity is relatively low, only a few band-shaped signals are clearly visible. In the right nucleus, the signal intensity is much higher. Such nuclei do not occur 70 min after heat shock. Accordingly, we assume that this nucleus exhibits a later pattern. For a detailed analysis of the dynamics of replication in the first 20 min of the S phase, we focused on region 4F-6A of chromosome X (boxed in Fig. 4a). The comparison of three consecutive patterns revealed that the paired signals in regions 4F9-10/5A1-2 and 5C1-2/5D develop sequentially from weaker diffuse signals emerging at the very beginning of the S phase (Fig. 4b–d). At later stages, the signals become brighter and larger and accumulate as thin bands. This finding clearly shows that the replication initiation in different chromatids takes place gradually in these regions.

**Fig. 5:**
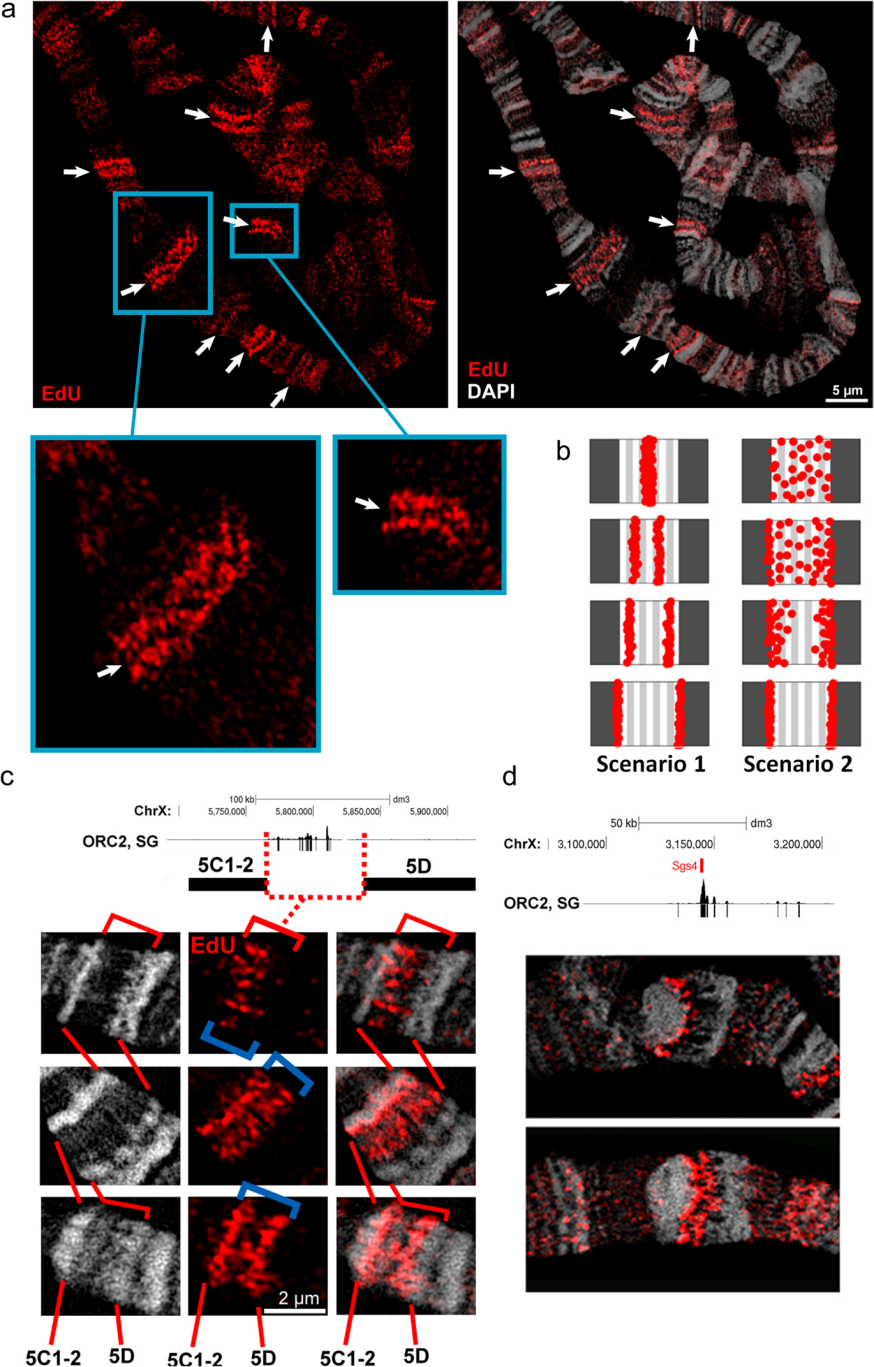
Two scenarios may induce similar replication signal pairs at rb-band edges during early replication. **a** Multiple EdU signal pairs of similar intensity are present along the polytene chromosomes. **b** Two hypothetical scenarios may cause these patterns. Paired signals may derive from replication forks diverging from very efficient origins, at which most DNA strands initiate replication synchronously (scenario 1). Replication initiation may also occur on different DNA strands at various origins, but due to the rapid movement of replication forks along the chromosome axis inside of the INTs, and owing to a sharp slowdown of the movement of replication forks along the chromosome axis inside the compact bands, the replication signals become concentrated on both edges of the INTs and thus appear also paired (scenario 2). **c** INT 5C1-2/5D illustrates scenario 2. The INT contains multiple ORC2-binding sites that predominate on the left side of the INT. The earliest detectable EdU pattern in this INT represents diffuse signals dominant in the left half of the INT (top). The two bottom patterns reflect the gradual accumulation of signals at the edges of the boundary rb-bands 5C1-2 and 5D. **d** The 3C locus illustrates scenario 1 (see Supplementary Figure 7 for details). The specimen was prepared from *Hsp70-CycE; SuUR* larvae, 90 min after heat shock

In most nuclei, symmetric bands of double signals are characteristic replication patterns in the early but not the earliest replication stage (Figs. 4 and 5a, Supplementary Figs. 7 and 8). Two hypothetical scenarios may be responsible for this pattern. First, double bands may reflect bidirectional replication forks moving in opposite directions from efficient origins in between. Namely, the signals look like a clear band perpendicular to the chromosome axis at the origin site and generate two distinct replication bands moving apart from each other. Second, replication initiation may also arise at various origins on different DNA strands but because of rapid replication fork movement followed by deceleration inside the compact bands, the replication signals become concentrated at the rim of highly condensed chromatin (rb-bands) and thereby appear paired (Fig. 5b). Consequently, both scenarios may result in similar paired signals. To clarify which scenario occurs predominantly in polytene chromosomes, we analyzed such double signals during progression in several individual regions by 3D-SIM.

The increased resolution allowed detecting many EdU signals scattered in the INT of region 5C/5D of the top chromosome X in Fig. 5c. Note, that the distribution of signals predominates at the left side of the INT, enriched in ORC2-binding sites mapped by chromatin immunoprecipitation (data from Sher et al. 2012). On the middle X chromosome, the entire INT is labeled, and the highest signal intensities levels correspond to the mirrored signals outside. On the bottom chromosome, the mirrored signals are mainly concentrated at the edges. Obviously, the three chromosomes represent the progression of replication forks initiated inside the INT toward the edges of boundary bands. Similar patterns were evident in regions 47D-48C and 41F-43B1-2 (Supplementary Fig. 8), i.e., during earlier stages, the signals are distributed uniformly within the INTs, but at later stages, they concentrate at the edges.

At locus 3C, we found a very thin bright signal at the very beginning of S phase. This is an example of a scenario with one efficient origin rather than a broad initiation zone (Fig. 5d). This early replication signal colocalizes with the *Sgs4* gene, forming a puff at the end of the third instar (Korge 1975; Supplementary Fig. 7c). Besides, at this locus, also a group of short genes occur, which are highly expressed in salivary glands (Supplementary Fig. 7d). All of them become activated by the hormone ecdysone, and their products are responsible for the secretion of the salivary gland (data from FlyBase database). That is, it is a group of tissue-specific genes. The distribution of ORC2-binding sites (Sher et al. 2012) shows that a small group of tissue-specific origins lie near this group of genes. The highest ORC2 peak corresponds to the *Sgs4* gene. Supplementary Fig. 7c demonstrates that the *Sgs4* FISH signal is flanked by EdU signals at the very early replication stage (paired signals occur). Thus, locus 3C demonstrates an example of scenario 1 (Fig. 5b). But even in this case, a cluster of several potential origins is evident. In general, the second scenario is much more common within the chromosomes.

In short, we draw the following conclusions. At the very beginning of the S phase, replication is not uniform at different sites. There are regions with distinct or diffuse EdU signals. Moreover, the number of diffuse signals varies among different INTs. The signal number per chromatid and signal size increase with time, indicating gradual replication initiation of different origins within the INT. Replication is initiated spatially stochastically inside the INTs. The activated replication forks pass quickly through the entire INTs in both directions to accumulate at INT borders. This finding is in good agreement with the notion that INTs match replication initiation zones with multiple potential origins.

### Replication spreads from the INTs into rb-bands devoid of ORC2 sites

**Fig. 6:**
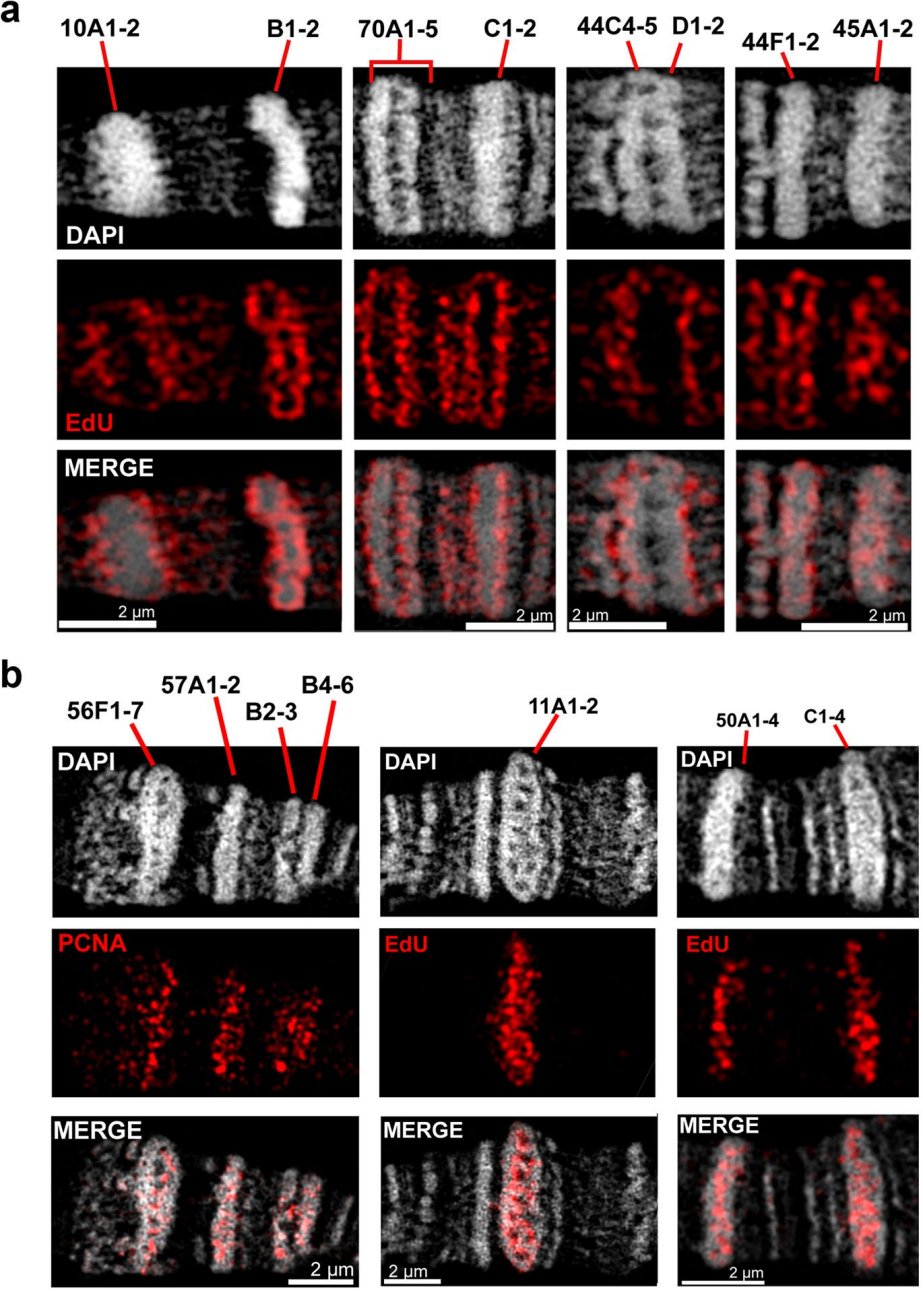
In the middle and late S phase, replication spreads from INTs into rb-bands. **a** Representative EdU labeling 3 h after heat shock (~100 min after S-phase initiation). At this middle-S-phase stage, most signals are concentrated along the borders of compact bands as seen in regions of chromosomes X (10A1-2 and B1-2), 3L (70A1-5 and C1-2), and 2R (44C1-2, D1-2, F1-2, and 45A1-2). The majority of EdU signals cover compact bands, but many signals are still visible between the rb-bands. At 10A1-2 borders, characteristic distortions of the band contour are noticeable. **b** During late-S-phase replication (not the induced S phase, *Hsp70-CycE; SuUR* larvae, without heat shock), the EdU signals are mainly concentrated inside the thickest compact rb-bands. In chromosome 2R region 56F-57B, PCNA staining is present inside intercalary-heterochromatin regions 56F1-7, 57A1-2, 57B1-2, and B4-6 (left). EdU signals occur in intercalary-heterochromatin bands 11A1-2 (middle), 50A1-4, and 50C1-4 (right)

Three hours after heat shock, most EdU signals accumulate at the borders of compact bands where characteristic distortions of the band contours emerge (Fig. 6a and Supplementary Fig. 9). This phenomenon is especially obvious in bands 70C and 10A (Fig. 6a). Supplementary Fig. 4b shows bands 10A and 10B from six nuclei from four preparations demonstrating earlier replication patterns. In none of them, such edge abnormalities occur. Thus, the distortions seem to be caused by the replication process.

Two very thick bands (50A and 50C) belong to intercalary heterochromatin (>200 kbp) and represent mainly ruby chromatin. They form prominent topologically associating domains (TADs) in salivary gland cells (Eagen et al. 2015) and lack any ORC2 sites. These bands feature bright EdU signals along their contour but not inside (Supplementary Fig. 10a). The thick rb-bands 21D1-2 and 22A1-2 behave likewise (Supplementary Fig. 10b).

In region 48D-49A, a group of pronounced rb-bands is located (Supplementary Fig. 9). The bands also represent mainly ruby chromatin and lack ORC2 sites. They form well-pronounced but smaller TADs in the salivary glands. The biggest (48E1-2) is ~100 kbp.

Not all rb-bands concentrate the EdU signal along their surfaces. In some preparations, band 50D appears as a distinct band, while on others, it occurs as swollen chromatin (a puff) implying high transcription intensity. Band 50D contains a low amount of ruby chromatin, and DAPI staining showed that it is significantly less condensed and is completely labeled by EdU throughout the entire volume (Supplementary Fig. 9). Hence, band 50D is not a typical rb-band. In terms of replication, it behaves differently from most rb-bands.

Overall, it can be concluded that uniformly compact rb-bands devoid of ORC2 sites are replicated from edges to the middle and that bands with complex organization and internal ORC2 sites can initiate replication inside.

The analysis of nuclei during late replication on preparations of wild-type and *hsp70-CycE; SuUR* larvae without heat shock revealed that the signals lie in the middle of very thick intercalary heterochromatin bands (Fig. 6b). Supplementary Figure 10 represents two examples of replication progression within thick bands at successive stages of middle and late S phase.

### Computer simulation confirms that dissimilar initiation rates may explain the different early replication patterns

**Fig. 7:**
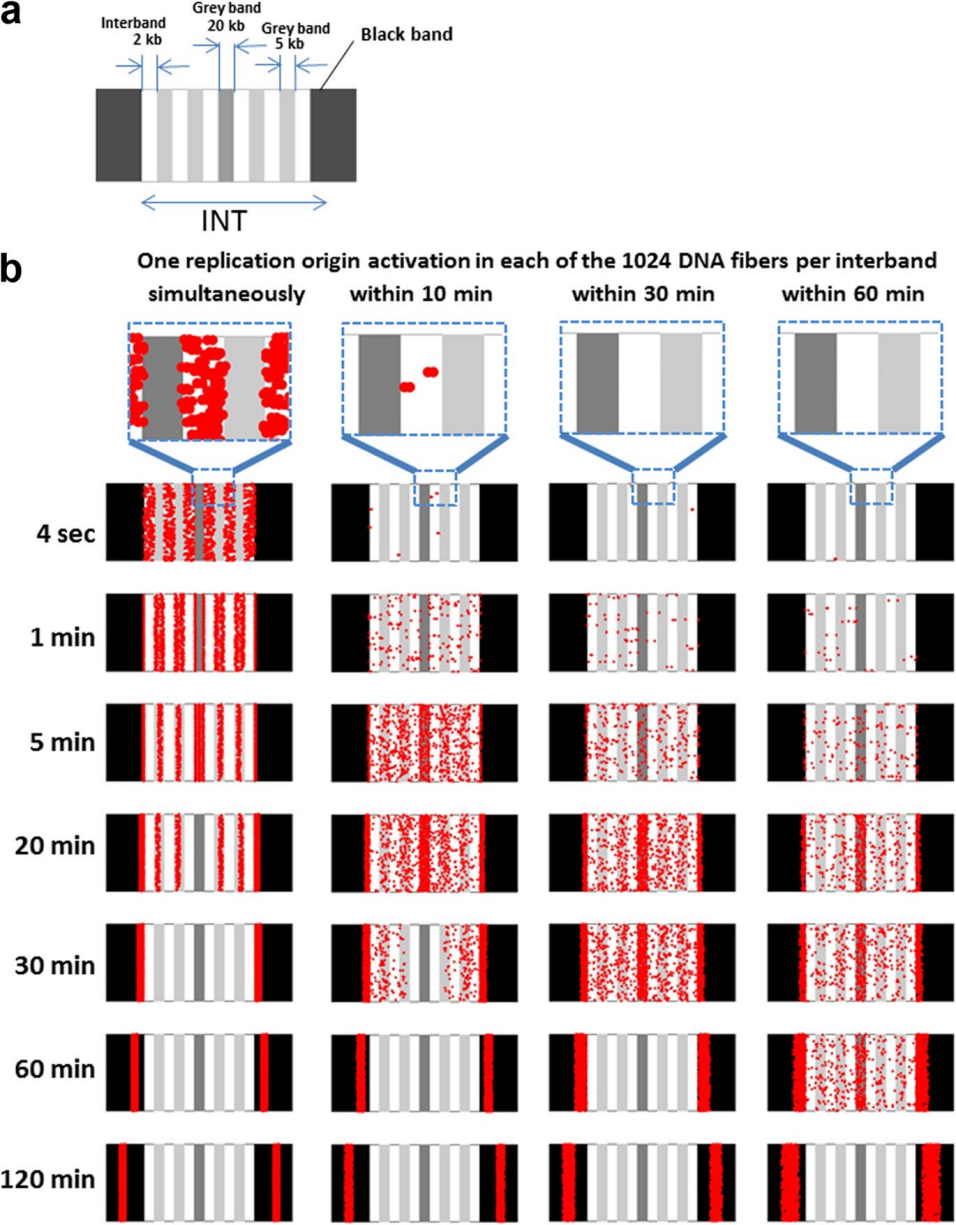
A computer simulation of replication progression in a 52 kbp INT localized between two rb-bands and composed of alternating interbands and gray bands of different compactness. **a** The INT includes six interbands and five gray bands. All bands were drawn to be of equal width along the chromosome axis but contain different DNA amounts. **b** In each of the 1024 DNA fibers, only one single replication origin activates at a random position within an interband. Then, each origin generates two replication forks running in opposite directions (visualized as red points) at a speed of 2 kbp/min. Four initialization scenarios were simulated (from left to right): One origin per chromatid may become activated in all chromatids simultaneously, within 10, 30, or 60 min. The replication fork distribution progresses from INTs toward rb-bands in both directions and is shown after 4 s to 120 min (from top to bottom)

To test the idea that different replication initiation rates are the main reason for the dissimilar earlier replication patterns observed in different INTs of polytene chromosomes, we performed the computer simulation of replication in a model chromosome fragment consisting of one INT limited by two rb-bands (Fig. 7, see “2” for details).

According to the model, a single replication origin activates in each chromatin fiber within interbands at a random position and gives rise to two replication forks, which are represented by red dots moving away from the origin along the chromosome axis at a rate constant for each type of structure. In Fig. 7, we consider four replication initiation scenarios: (1) all 1024 origins of the INT fire simultaneously; (2) the origins are activated gradually at a rate of ~10 origins per minute, that is, all 1024 origins are activated within 10 min; (3) 1024 origins are activated within 30 min; and (4) 1024 origins are activated within 60 min. The scenarios reflect the various experimentally observed replication initiation patterns in different INTs.

Replication fork speed was assumed to be 2 kbp/min. Therefore, the replication rate (the rate of the red point movement) along the interband was *V* = 1 interband/min. The relative speed was *V*/5 in a gray band, *V*/20 in the middle gray band, and *V*/50 in a black band. The distribution of red points in the INT after 4 s and 1, 5, 20, 30, 60, and 120 min is shown from top to bottom in Fig. 7.

We noticed the following trends: With synchronous initiation of replication, within 1 min, all signals gravitate toward the bands. There are bright signals in the form of stripes from the very beginning because the signals from the interbands are concentrated in the bands that contain more DNA and the probability of encountering a signal there is higher. After 30 min, an INT with a length of ~50 kbp completes replication, and the signals become appreciably concentrated at the edges of the condensed thick bands.

When the initiation of replication is prolonged, we see fewer ordered signals at all stages. A high concentration of signals is present in the more compact middle gray band and at the edges of the black bands, i.e., in the bands with more DNA per unit of chromosome length. The longer replication initiation takes, the longer are diffusely scattered signals present throughout the INT.

Thus, different rates of replication initiation explain very well the dissimilar replication patterns in the different INTs of polytene chromosomes.

## Discussion

### *Drosophila* polytene chromosomes are well-suited for deciphering replication

In this work, we demonstrated that polytene chromosomes are well-suited to investigate the very early replication parameters of *Drosophila* that are probabilistic. In these chromosomes, more than 1000 DNA filaments are arranged in parallel with a distinct pattern of thick and thin bands denoting TADs (Zhimulev et al. 2014; Eagen et al. 2015; Ulianov et al. 2016; Stadler et al. 2017; Kolesnikova 2018). Highly stretched chromosomes allow the direct visualization of differently compacted chromatin along the chromosome axis. At the beginning of the S phase, the distribution and density of replication signals reflect the probability of replication initiation. The replication initiation zones correspond to INTs, and EdU signal density within the INTs reflects their efficiency. Moreover, the efficiency here is not only the probability of activation during the cell cycle but also the probability of activation per time unit during early S phase. The distribution of early origins in cultured cells exposed to hydroxyurea (MacAlpine et al. 2010) is in good agreement with our reasoning about the dissimilar origin efficiency grades among different INTs.

We showed that ectopic S-phase induction is useful for investigating early replication in *Drosophila* polytene chromosomes. The analysis of replication in salivary glands of wild-type larvae grown under standard conditions (24–25 °C) did not permit identifying very early replication patterns because the stages preceding continuous labeling take several minutes. Other authors used temperature reduction, the *Giant* mutation, and an analysis of other *Drosophila* species to find a model system that would allow more detailed research on the earliest S-phase patterns (Roy and Lakhotia 1979, 1981; Mishra and Lakhotia 1982). The synchronization with FdU induced the accumulation of later S-phase stages in salivary gland cells (Achary et al. 1981). To study the stages in detail, which are normally too short in *D. melanogaster*, we developed a system based on ectopic S-phase induction. The use of the *hsp70-CycE* transgene (Knoblich et al. 1994) helped to induce S phases in many salivary gland nuclei with a time shift of no more than 20 min. We demonstrated that the replication patterns are identical between the induced and normal S phases. The S phases caused by ectopic cyclin E expression differ from those without induction in diploid cells. Here, due to the transcription/replication conflict, the initiation events occur at the wrong sites (Teixeira and Reed 2017; Macheret and Halazonetis 2018). One would expect similar differences between the induced S phase and the normal one in polytene chromosomes, but we did not find examples of ectopic initiation of replication at the beginning of the S phase. The correct early replication initiation during ectopic S phase in salivary gland may occur due to the specificity of the endocycle regulation, because the amount of MSM2-7 complex components is lower than in diploid cells (Maqbool et al. 2010). This could prevent covering all chromosomes with MSM2-7, and thus preventing initiation at wrong places.

Despite the fact that the early S phase does not differ from the normal one during the induction of replication in our system, the middle and late S phases appear to be impaired. The reason may be that in contrast to diploid cells, the E2F (transcription regulator of many genes involved in the S-phase progression) acts upstream of cyclin E. In particular, the synthesis of ribonucleotide reductase is under control of E2F. It can be assumed that the ectopic expression of cyclin E triggers S phase without activating some genes necessary for the complete passage of the S phase (Edgar et al. 2014; Kim et al. 2021; Dimova and Dyson 2005). Possibly, that the cells enter the induced S phase with a limited amount of nucleotides.

### Replication initiation is stochastic in polytene chromosomes

During the 1970s, DNA replication studies on *Drosophila* polytene chromosomes were conducted via ^3^H-thymidine incorporation. This method allowed researchers to draw quantitative conclusions about the intensity of label inclusion after silver grain counting. Various authors have identified up to seven categories of labeling patterns and arranged them chronologically (Rodman 1968; Kalisch and Hägele 1973; Roy and Lakhotia 1979, 1981; Achary et al. 1981; Mishra and Lakhotia 1982). The labels have been detected in decondensed areas (called interbands in the cited papers, corresponding to our INTs) in three early stages representing low, medium, and heavy interband patterns. During these stages, a sequential increase in the silver grain number occured. It has turned out that the regions differ in the rate of ^3^H thymidine incorporation. There are regions in which many silver grains arose already during the first minutes of the S phase. Circa 40 such areas have been observed. After these stages of early discrete labeling, stages of continuous labeling followed. Besides, the stages differed from each other in labeling intensity, showing medium or heavy the continuous labeling. In the last three stages, the discrete labeling decreased in number and signal intensity (heavy, medium, and low discontinuous labeling), indicating the exit of most replicons from replication (Mishra and Lakhotia 1982). Our results completely match these earlier findings, namely, that during the first hour after S-phase induction, new replicons are switched on, as revealed by a signal intensity increase. Additionally, various chromosomal regions manifest different dynamics of replication initiation.

The detection of a “continuous labeling” stage was due to the low resolution of autoradiography (~1 μm), which is determined by the size of the emulsion grain and the path length of β particles after tritium decay. Even in larvae with a delayed S phase, those authors have observed a “continuous” coverage of chromosomes with a signal present already 10 min after the S-phase beginning.

The use of fluorescent labeling in combination with WF microscopy allows to identify signal gaps in the thickest bands at the stage of continuous labeling, but the maximum resolution does not exceed thick band/INT sizes (Kolesnikova et al. 2018). In the present work, we applied super-resolution microscopy and reached for the first time a resolution visualizing single signals representing multiple replication patterns.

The model of stochastic replication initiation in polytene chromosomes was first proposed by Lakhotia and Sinha (1983). After an analysis of fibrils in partially lysed chromosomes, they concluded that initiation on different DNA strands is asynchronous. Our results fully support this model.

Stochastic regulation of replication kinetics is a fundamental feature of eukaryotes and is conserved from yeast to humans (Wang et al. 2021). Origin efficiency usually refers to the probability of activation during the cell cycle. Nevertheless, there is accumulating evidence that this parameter is more complex. It can be regarded as the competition between origins for activation, where more efficient origins have a higher activation probability at the S-phase start. In contrast, after the release of limiting factors, a new pool of origins becomes competitive. The observation that on average, 9% of late-initiating origins initiate replication in the early S phase led to the idea that origin activation probability determines all replication timing (Wang et al. 2021). The results of our work are in good agreement with these new ideas about the gradual initiation of replication across the genome.

### *Replication in Drosophila and mammals: similarities and differences*

The general genome organization differs between *Drosophila* and mammals. The *Drosophila* genome is almost an order of magnitude more compact. While the mammalian genome contains megabase scale chromatin domains and TADs well coinciding with replication domains, the alternation of shorter domains is present in the *Drosophila* genome. These are open chromatin domains with a median size of ~30 kbp attracting more than 90% of ORC-binding sites. Closed-chromatin domains contain predominantly silent tissue-specific genes and almost no ORC-binding sites (Zhimulev et al. 2014; Kolesnikova et al. 2018). INTs representing the replication initiation zones in *Drosophila* match those in mammals very well in size and many properties. An important difference is that the replication initiation zones in mammals lie predominantly in long intergenic regions (Lebofsky et al. 2006; Petryk et al. 2016). Instead, in *Drosophila*, they are situated in a set of short intergenic regions alternating with housekeeping genes (Kolesnikova et al. 2018). In mammals, the characteristic replicon size is >100 kbp (Edenberg and Huberman 1975; Berezney et al. 2000; Lebofsky et al. 2006). In *Drosophila*, this size is similar, but almost coincide with the median size of silent domains. Apparently, these silent domains of chromosome arms (corresponding to rb-bands of polytene chromosomes) are replicated mostly passively, that is, by replication forks coming from neighboring INTs (Kolesnikova et al. 2018).

We suppose that the patterns of replication initiation that we observed in polytene chromosomes are universal because they are consistent with the recent finding that replication initiation occurs stochastically in space and time, and that replication initiation events are distributed across broad initiation zones consisting of many initiation sites, whereas the dynamics is heterogeneous (Su et al. 2020; Wang et al. 2021).

## Supplementary Information

ESM 1 (DOCX 4.15 MB)

Supplementary Movie 1 : Early replication in wild-type chromosome 2L. (AVI 7.47 MB)

Supplementary Movie 2 : Early replication in the distal part of chromosome 2L shown in Figure 5. (AVI 6.51 MB)

Supplementary Movie 3 : Enlarged INT 21C1-2/21D1-2 presented in Figure 5. (AVI 8.45 MB)

Supplementary Movie 4 : Enlarged INT 21D1-2/21E1-2 depicted in Figure 5. (AVI 6.50 MB)

Supplementary Movie 5 : Enlarged INT 21E1-2/22A1-2 displayed in Figure 5. (AVI 8.47 MB)

Supplementary Fig. 1 **3D-SIM allows detecting** **more structural details in comparison with wide-field WF microscopy. (a)** Comparison of early, middle, and late S-phase replication patterns of region 46A-48A (chromosome 2R of wild-type larvae) imaged by WF microscopy and 3D-SIM. The increased resolution achieved by 3D-SIM is especially notable in the enlarged regions (rectangles) on the right. The fluorescence EdU, WF, and SIM images were merged with phase contrast (Phaco) and fluorescence DAPI images, respectively. **(b)** The enlarged region showing very early replication demonstrates the significantly increased resolution achieved by 3D-SIM compared to WF microscopy. Hsp70-CycE; SuUR larvae, 90 min after heat shock. **(c)** Ortho-view of the distal region of wild-type chromosome 2L in early and late S-phase stages. In each chromosome, two different ortho-view positions are visualized. Note that the chromosomes keep their 3D shape as indicated in the x-z and y-z cross-sections. EdU is present throughout the entire chromosome volume (JPG 2.47 MB)

Supplementary Fig. 2 **The effect of chromosome stretching on the replication signal shape in** ***Hsp70-CycE; SuUR*** **larvae, 90 min after heat shock.** **(a)** Asegment of chromosome 2R including regions 46A-47D, from two squash preparations (Slides 1 and 2). On Slide 1, region 46A-47A is stretched threefold less than in the two examples from Slide 2. This means that no strong chromatin fiber displacement happens along the chromosome axis. Via the chromosome stretching, polytene chromosome band/interband structure becomes more obvious, and the EdU signals spread more within the thin bands. **(b)** Stronger chromosome stretching induces flattening, as evidenced by the ortho-view of the chromosomes in (**a)**. **(c)** At higher magnification, a comparison of two selected chromosome regions from the same three nuclei as in **(a)** reveals a difference in the shape of the signals between the less stretched (top) and more stretched (two lower) chromosomes. **(d) **The scheme explaining the impact of chromosome stretching on the apparent local replication pattern. Stretching visualizes compact and less compact regions along the chromosome axis. EdU labeling becomes visible at different grey band positions in a chromatid, with the formation of a clear-cut band/interband structure. In less stretched chromosomes, the spatial interactions between chromosome regions within the INT are preserved. Replication sites from different chromatids stay together and form replication factories. The band/interband pattern is not discernible on the less stretched chromosomes (JPG 1.20 MB)

Supplementary Fig. 3 Replication patterns in wild-type (WT) larvae without heat shock and in *Hsp70-CycE; SuUR* larvae after S-phase induction by heat shock. **(a)** In contrast to WT, *Hsp70-CycE; SuUR* larvae with the heat shock–induced S phase (90 min after heat shock) feature an additional round of polytenization leading to larger and betterstructured polytene chromosomes. **(b) **The early replication patterns are similar on the same slide but may vary among different slides as exemplified in the distal part of chromosome 2L. The red arrow points to a varied signal in INT 21D1-2/21E1-2. **(c) **The replication patterns are similar between nuclei from WT larvae and *Hsp70-Cyc; E SuUR* larvae 60 to 180 min after heat shock (induced (IND) S phases), as indicated by the patterns of early, middle, and late S phases of region 56A-57B on chromosome 2R. Late replication patterns were noted in cells that were already in the S phase before heat shock (JPG 3.30 MB)

Supplementary Fig. 4 Reproducibility of the polytene chromosome morphology and early replication patterns in the 10A-11A region of chromosome X as detected by the EdU incorporation assay and PCNA immunolabeling. (**a**) The seven photographs originate from three preparations (Slides 1, 2, and 4; the slide numbering is identical to that in Supplementary Figures 2 and 3). *Hsp70-CycE SuUR* larvae, 90 min after heat shock. On Slide 1, the signals are arranged as bright lines perpendicular to the chromosome axis but as dispersed spots on Slides 2 and 4. On Slide 2, the chromosome is more stretched than on Slide 1. (**b**) Morphology of 10A1-2 and 10B1-2 bands (enlarged fragments of chromosomes stained with DAPI from a) (JPG 1.61 MB)

Supplementary Fig. 5 Simultaneous detection of very early replication (EdU) and the active RNA polymerase II variant phosphorylated at serine 2 (RNAPIIser2ph). (**a**) Nucleus at the very early replication stage in *Hsp70-CycE; SuUR* larvae, 90 min after heat shock. Ca. 30 bright EdU sites are present, but significantly more active transcription signals occur. Some of them colocalize, as exemplified for region 47 of chromosome 2R (rectangle in the phase contrast image). (**b**) Schema of the INT between rb-bands 47A1-2 and 47B4-5. The genomic coordinates of the rb-bands (Kolesnikova et al. 2018), ORC2 binding sites (Sher et al., 2012), “Early origins in Kc” (MacAlpine et al., 2010) and of the lola 3’ and lola 5’ FISH probes are indicated. Data on gene expression in salivary glands (SG) of third instar larvae originate from the ModEncode project. **(c)** At locus 47AB efficient early replication initiation starts in the vicinity of the promoter of the actively transcribed *lola *gene. The EdU signal partially coincides with the probe corresponding to the 5’ end of the gene, but lies distant from the probe corresponding to its 3 'end (JPG 3.10 MB)

Supplementary Fig. 6 Localization of polytene chromosome bands in the distal part of chromosome 2L on the *D. melanogaster* genome map. **(a) **Mapping of rb-bands by an algorithm developed by Kolesnikova et al. (2018). The optical section of a 3D-SIM image stack depicts a fragment of chromosome 2L stained with DAPI. Four very compact bands, 21C1-2, D1-2, E1-2, and 22A1-2, are marked. Below, on the scale of the genomic map, the predicted genomic coordinates of these bands are given, as is a distribution of chromatin properties in the respective region, enabling one to determine the coordinates. The 4-chromatin-states model postulates that all four bands are enriched with ruby chromatin and can be assigned to rb-bands. The presence of the CHRIZ protein in four cell cultures (data from the ModEncode project) is an important marker of polytene chromosome interbands and accordingly is a marker of the band boundaries. Regions of SuUR protein enrichment in SG cells [data from Maksimov et al. (2014)] are also a good marker of rb-bands and their boundaries. A fragment of the contact matrix constructed according to the results of Hi-C analysis in SG cells (Eagen et al. 2015) suggests that the highlighted regions correspond to well-pronounced TADs. This finding further proves that these regions match rb-bands. **(b)** Mapping of bands 21C4 and 21C6 in the chromosome 2L fragment. These bands are less compact than the rb-bands highlighted in **(a)**. Above are representative photographs of *in situ* hybridization assays of P inserts from the BDGP project (Spradling et al. 1999) mapped to INT 21C1-2/21D1-2. The coordinates of the inserts are described by Spradling et al. (1999), and the images were kindly provided by Tod Laveltry. These images helped to approximately localize 21C4 and 21C6 bands on the genomic map (vertical red dotted lines) and revealed the absence of ruby chromatin. The distribution of red chromatin (active promoters) according to the model of 9-chromatin states (Kharchenko et al. 2011) and of the CHRIZ protein in four cell cultures allowed us to predict the most probable coordinates of the interband. The distribution of the ORC2 protein in salivary glands (SGs) is based on data from Sher et al. (2012). The distribution of newly synthesized DNA in the presence of hydroxyurea (“Early origins”) in S2 cells was published by MacAlpine et al. (2010). “Expression SG” represents the gene expression level in SGs of third instar larvae. Color coding represents expression levels (data from the ModENCODE project) (JPG 2.89 MB)

Supplementary Fig. 7 **The** ***Sgs4*** **gene** **region is an example of efficient local replication initiation** (Scenario 1 in figure 5b). **(a) **At very early replication paired signals are not yet visible on a X chromosome fragment, but slightly later they occur **(b)**. The red frames indicate the 3C locus of both X chromosomes.** (c) **Simultaneous detection of early replication (stage of paired EdU signals) and the *Sgs4* gene via FISH at locus 3C. **(d) **Part of the genomic map of the *Sgs4* gene region containing a cluster of genes actively expressed in third larval instar salivary glands. A cluster of ORC2 sites detected in salivary glands is not typical for the Kc cell line. The coincidence of the highest ORC2 peak with the *Sgs4* gene is obvious (JPG 1.62 MB)

Supplementary Fig. 8 **Replication dynamics at the earliest S-phase stages within INTs 90 min after heat shock.** **(a) **Between rb-bands 47D1-6 and 48C1-2, at an earlier stage (top), three INTs (denoted by red brackets) contain multiple point signals evenly distributed across the INTs. At a later stage (bottom), the same INTs accumulate signals along the edges of the INT, but also many EdU signals are still present throughout the entire INT, as especially noticeable in INT 4A1-2/C1-2. **(b) **A similar dynamics of transitions from diffuse signals to signals accumulated at the INT edges occur in the INTs surrounding rb-band 42A1-2 (JPG 1.61 MB)

Supplementary Fig. 9 Only pronounced rb-bands replicate from their periphery to the interior. **(a)** An optical slice with enlarged regions of a 3D-SIM image stack of region 48D-50D (chromosome 2R) stained by DAPI and EdU incorporation 3 h after heat shock. Within the enlarged regions, rb-bands with this replication pattern can be identified based on genomic coordinates (**b**, **c**). **(b)** Genomic coordinates of the regions showing compact bands (black rectangles) can be assigned to rb-bands because according to the 4-chromatin-states model (Zhimulev et al. 2014), regions enriched with ruby chromatin (magenta) are evident. Color coding illustrates expression levels in salivary glands (Expression SG) of third instar larvae in the respective regions (data from the ModENCODE project). The presence of the CHRIZ protein noted in four cell cultures (data from the ModEncode project) is an important marker of polytene chromosome interbands and accordingly is an indicator of band boundaries. Fragments of a Hi-C contact matrix originating from SG cells [according to Eagen et al. (2015)] reveal that the highlighted regions correspond to well-pronounced TADs and compact rb-bands. The distribution of ORC2 protein in SGs is based on data from Sher et al. (2012) **(c)** Characteristic morphology of regions 49F and 50D. In region 49F, the compact band is close to the left-hand loose material; a clear-cut interband cannot be visualized either by aceto-orcein staining or by electron microscopy. Instead, band 50D1-2 may form a puff pointing to a high level of expression (JPG 1.46 MB)

Supplementary Fig. 10 **Two examples of mid-late S phase replication dynamics.** **(a) **Region 50A-C.** (b) **Region 21D-22A (JPG 1.54 MB)

Supplementary Text 1 **A replication simulation for a polytene chromosome fragment.** The computer program was written in the Delphi Pascal language (DOCX 21.2 kb)

## Abbreviations

3D-SIM: Spatial structured illumination microscopy
BSA: Bovine serum albumin
DAPI: 4′,6-Diamidino-2-phenylindole
EdU: 5-Ethynyl-2′-deoxyuridine
FISH: Fluorescence in situ hybridization
h: Hours
min: Minutes
INTs: Intervals between rb-bands
ORC: Origin recognition complex
PBS: Phosphate-buffered saline
PCNA: Proliferating cell nuclear antigen
PCR: Polymerase chain reaction
Rb-bands: Polytene chromosome bands characterized by the presence of “ruby” chromatin according to Zhimulev et al. (2014) and Kolesnikova et al. (2018)
RNAPIIser2ph: RNA polymerase II phosphorylated at serine 2
SSC: Standard saline citrate
TAD: Topologically associating domain
WF: Wide-field

## References

[CR1] Achary PM, Majumdar K, Duttagupta A, Mukherjee AS (1981). Replication of DNA in larval salivary glands of *Drosophila* after in vivo synchronization. Chromosoma.

[CR2] Anglana M, Apiou F, Bensimon A, Debatisse M (2003). Dynamics of DNA replication in mammalian somatic cells: nucleotide pool modulates origin choice and interorigin spacing. Cell.

[CR3] Baddeley D, Chagin VO, Schermelleh L (2010). Measurement of replication structures at the nanometer scale using super-resolution light microscopy. Nucleic Acids Res.

[CR4] Bechhoefer J, Rhind N (2012). Replication timing and its emergence from stochastic processes. Trends Genet.

[CR5] Beermann W (1972). Chromomeres and genes. Results Probl Cell Differ.

[CR6] Belyaeva ES, Goncharov FP, Demakova OV et al (2012) Late replication domains in polytene and non-polytene cells of *Drosophila melanogaster*. PLoS One 7. 10.1371/journal.pone.0030035 10.1371/journal.pone.0030035 PMC3254639 22253867

[CR7] Belyaeva ES, Zhimulev IF, Volkova EI (1998). Su(UR)ES: a gene suppressing DNA underreplication in intercalary and pericentric heterochromatin of *Drosophila melanogaster* polytene chromosomes. Proc Natl Acad Sci USA.

[CR8] Berezney R, Dubey DD, Huberman JA (2000). Heterogeneity of eukaryotic replicons, replicon clusters, and replication foci. Chromosoma.

[CR9] Besnard E, Babled A, Lapasset L (2012). Unraveling cell type-specific and reprogrammable human replication origin signatures associated with G-quadruplex consensus motifs. Nat Struct Mol Biol.

[CR10] Bleichert F, Botchan MR, Berger JM (2017) Mechanisms for initiating cellular DNA replication. Science 355. 10.1126/science.aah6317 10.1126/science.aah6317 28209641

[CR11] Boldyreva LV, Goncharov FP, Demakova OV (2017). Protein and genetic composition of four chromatin types in Drosophila melanogaster cell lines. Curr Genomics.

[CR12] Borowiec JA, Schildkraut CL (2011). Open sesame: activating dormant replication origins in the mouse immunoglobulin heavy chain (Igh) locus. Curr Opin Cell Biol.

[CR13] Chagin VO, Casas-Delucchi CS, Reinhart M (2016). 4D Visualization of replication foci in mammalian cells corresponding to individual replicons. Nat Commun.

[CR14] Demakov SA, Vatolina TY, Babenko VN (2011). Protein composition of interband regions in polytene and cell line chromosomes of Drosophila melanogaster. BMC Genomics.

[CR15] Demakova OV, Demakov SA, Boldyreva LV (2020). Faint gray bands in Drosophila melanogaster polytene chromosomes are formed by coding sequences of housekeeping genes. Chromosoma.

[CR16] Demczuk A, Gauthier MG, Veras I (2012). Regulation of DNA replication within the immunoglobulin heavy-chain locus during B cell commitment. PLoS Biol.

[CR17] Dijkwel PA, Wang S, Hamlin JL (2002). Initiation sites are distributed at frequent intervals in the Chinese hamster dihydrofolate reductase origin of replication but are used with very different efficiencies. Mol Cell Biol.

[CR18] Dileep V, Gilbert DM (2018). Single-cell replication profiling to measure stochastic variation in mammalian replication timing. Nat Commun.

[CR19] Dimova DK, Dyson NJ (2005). The E2F transcriptional network: old acquaintances with new faces. Oncogene.

[CR20] Duronio RJ, O’Farrell PH (1995). Developmental control of the G1 to S transition in Drosophila: cyclin Eis a limiting downstream target of E2F. Genes Dev.

[CR21] Eagen KP, Hartl TA, Kornberg RD (2015). Stable chromosome condensation revealed by chromosome conformation capture. Cell.

[CR22] Eaton ML, Prinz JA, MacAlpine HK (2011). Chromatin signatures of the Drosophila replication program. Genome Res.

[CR23] Edgar BA, Zielke N, Gutierrez C (2014). Endocycles: a recurrent evolutionary innovation for post-mitotic cell growth. Nat Rev Mol Cell Biol.

[CR24] Edenberg HJ, Huberman JA (1975). Eukaryotic chromosome replication. Annu Rev Genet.

[CR25] Gong WJ, Golic KG (2006). Loss of Hsp70 in Drosophila is pleiotropic, with effects on thermotolerance, recovery from heat shock and neurodegeneration. Genetics.

[CR26] Gros J, Kumar C, Lynch G (2015). Post-licensing specification of eukaryotic replication origins by facilitated Mcm2-7 sliding along DNA. Mol Cell.

[CR27] Hamlin JL, Mesner LD, Lar O (2008). A revisionist replicon model for higher eukaryotic genomes. J Cell Biochem.

[CR28] Heichinger C, Penkett CJ, Bähler J, Nurse P (2006). Genome-wide characterization of fission yeast DNA replication origins. EMBO J.

[CR29] Herrick J (2011). Genetic variation and DNA replication timing, or why is there late replicating DNA?. Evolution.

[CR30] Kalisch WE, Hägele K (1973). Different DNA replication behavior of a tandem duplication in Drosophila melanogaster. Chromosoma.

[CR31] Khoroshko VA, Pokholkova GV, Levitsky VG (2020). Genes containing long introns occupy series of bands and interbands in Drosophila melanogaster polytene chromosomes. Genes (Basel).

[CR32] Kim M, Delos Santos K, Moon N-S (2021). Proper CycE-Cdk2 activity in endocycling tissues requires regulation of the cyclin-dependent kinase inhibitor Dacapo by dE2F1b in Drosophila. Genetics.

[CR33] Knoblich JA, Sauer K, Jones L (1994). Cyclin E controls S phase progression and its down-regulation during Drosophila embryogenesis is required for the arrest of cell proliferation. Cell.

[CR34] Kolesnikova TD (2018). Banding pattern of polytene chromosomes as a representation of universal principles of chromatin organization into topological domains. Biochemistry (Mosc).

[CR35] Kolesnikova TD, Demakov SA, Ivankin AV (2009). The mutation of the suppressor of underreplication gene does not affect the replication fork rate in the Drosophila melanogaster salivary gland polytene chromosomes. Dokl Biochem Biophys.

[CR36] Kolesnikova TD, Goncharov FP, Zhimulev IF (2018) Similarity in replication timing between polytene and diploid cells is associated with the organization of the Drosophila genome. PLoS One 13. 10.1371/journal.pone.0195207 10.1371/journal.pone.0195207 PMC5902040 29659604

[CR37] Kolesnikova TD, Posukh OV, Andreyeva EN (2013). Drosophila SUUR protein associates with PCNA and binds chromatin in a cell cycle-dependent manner. Chromosoma.

[CR38] Korge G (1975). Chromosome puff activity and protein synthesis in larval salivary glands of *Drosophila melanogaster*. Proc Natl Acad Sci U S A.

[CR39] Kozlova TU, Semeshin VF, Tretyakova IV (1994). Molecular and cytogenetical characterization of the 10A1-2 band and adjoining region in the *Drosophila melanogaster* polytene X chromosome. Genetics.

[CR40] Kutskova IA, Mamon LA (1995). The regression time of heat-shock puffs in the polytene chromosomes of *Drosophila melanogaster* as a criterion for assessing the effect of different stress exposures. Tsitologiia.

[CR41] Lakhotia SC (1974). EM autoradiographic studies on polytene nuclei of *Drosophila melanogaster*. 3. Localisation of non-replicating chromatin in the chromocentre heterochromatin. Chromosoma.

[CR42] Lakhotia SC, Sinha P (1983). Replication in *Drosophila* chromosomes. X. Two kinds of active replicons in salivary gland polytene nuclei and their relation to chromosomal replication patterns. Chromosoma.

[CR43] Lebofsky R, Heilig R, Sonnleitner M (2006). DNA replication origin interference increases the spacing between initiation events in human cells. Mol Biol Cell.

[CR44] Lefevre E (1976) A photographic representation and interpretation of the polytene chromosomes of *Drosophila melanogaster* salivary glands. In: The genetics and biology of *Drosophila*, Ashburner M, editor. Volume 1a. Academic Press, London, New York, pp 31–36

[CR45] Löb D, Lengert N, Chagin VO (2016). 3D replicon distributions arise from stochastic initiation and domino-like DNA replication progression. Nat Commun.

[CR46] Lubelsky Y, Sasaki T, Kuipers MA (2011). Pre-replication complex proteins assemble at regions of low nucleosome occupancy within the Chinese hamster dihydrofolate reductase initiation zone. Nucleic Acids Res.

[CR47] MacAlpine HK, Gordân R, Powell SK (2010). Drosophila ORC localizes to open chromatin and marks sites of cohesin complex loading. Genome Res.

[CR48] Macheret M, Halazonetis TD (2018). Intragenic origins due to short G1 phases underlie oncogene-induced DNA replication stress. Nature.

[CR49] Maksimov DA, Koryakov DE, Belyakin SN (2014). Developmental variation of the SUUR protein binding correlates with gene regulation and specific chromatin types in *D. melanogaster*. Chromosoma.

[CR50] Massey DJ, Koren A (2021) High-throughput analysis of DNA replication in single human cells reveals constrained variability in the location and timing of replication initiation. bioRxiv:2021.05.14.443897. 10.1101/2021.05.14.443897

[CR51] Maqbool SB, Mehrotra S, Kolpakas A (2010). Dampened activity of E2F1-DP and Myb-MuvB transcription factors in *Drosophila* endocycling cells. J Cell Sci.

[CR52] Méchali M (2010). Eukaryotic DNA replication origins: many choices for appropriate answers. Nat Rev Mol Cell Biol.

[CR53] Mesner LD, Valsakumar V, Cieslik M (2013). Bubble-seq analysis of the human genome reveals distinct chromatin-mediated mechanisms for regulating early- and late-firing origins. Genome Res.

[CR54] Mesner LD, Valsakumar V, Karnani N (2011). Bubble-chip analysis of human origin distributions demonstrates on a genomic scale significant clustering into zones and significant association with transcription. Genome Res.

[CR55] Meyer LR, Zweig AS, Hinrichs AS (2013). The UCSC Genome Browser database: extensions and updates 2013. Nucleic Acids Res.

[CR56] Miotto B, Ji Z, Struhl K (2016). Selectivity of ORC binding sites and the relation to replication timing, fragile sites, and deletions in cancers. Proc Natl Acad Sci U S A.

[CR57] Mishra A, Lakhotia SC (1982). Replication in *Drosophila* chromosomes. VII. Influence of prolonged larval life on patterns of replication in polytene chromosomes of *Drosophila melanogaster*. Chromosoma.

[CR58] Roy S, Ernst J, modENCODE Consortium (2010). Identification of functional elements and regulatory circuits by Drosophila modENCODE. Science.

[CR59] Moldovan G-L, Pfander B, Jentsch S (2007). PCNA, the maestro of the replication fork. Cell.

[CR60] Petryk N, Kahli M, d’Aubenton-Carafa Y (2016). Replication landscape of the human genome. Nat Commun.

[CR61] Posukh OV, Maksimov DA, Laktionov PP (2017). Functional dissection of *Drosophila melanogaster* SUUR protein influence on H3K27me3 profile. Epigenetics Chromatin.

[CR62] Powell SK, MacAlpine HK, Prinz JA et al (2015) Dynamic loading and redistribution of the Mcm2-7 helicase complex through the cell cycle. EMBO J 34(531–543). 10.15252/embj.201488307 10.15252/embj.201488307 PMC4331006 25555795

[CR63] Raghuraman MK, Winzeler EA, Collingwood D (2001). Replication dynamics of the yeast genome. Science.

[CR64] Rhind N, Yang SC-H, Bechhoefer J (2010). Reconciling stochastic origin firing with defined replication timing. Chromosome Res.

[CR65] Rodman TC (1968). Relationship of developmental stage to initiation of replication in polytene nuclei. Chromosoma.

[CR66] Roy S, Lakhotia SC (1979). Replication in *Drosophila* chromosomes: part II—unusual replicative behaviour of two puff sites in polytene nuclei of *Drosophila kikkawai*. Indian J Exp Biol.

[CR67] Roy S, Lakhotia SC (1981) Replication in *Drosophila* chromosomes. IV. Patterns of chromosomal replication in salivary gland polytene nuclei of *Drosophila nasuta*. Ind J Exp Biol 19:797–807

[CR68] Saura A (1986). Electron microscopic mapping of the second polytene chromosome of *Drosophila melanogaster*.

[CR69] Schermelleh L, Carlton PM, Haase S (2008). Subdiffraction multicolor imaging of the nuclear periphery with 3D structured illumination microscopy. Science.

[CR70] Schwaiger M, Stadler MB, Bell O (2009). Chromatin state marks cell-type- and gender-specific replication of the *Drosophila* genome. Genes Dev.

[CR71] Sher N, Bell GW, Li S (2012). Developmental control of gene copy number by repression of replication initiation and fork progression. Genome Res.

[CR72] Spierer A, Spierer P (1984). Similar level of polyteny in bands and interbands of *Drosophila* giant chromosomes. Nature.

[CR73] Spradling AC, Stern D, Beaton A (1999). The Berkeley *Drosophila* Genome Project gene disruption project: single P-element insertions mutating 25% of vital *Drosophila* genes. Genetics.

[CR74] Stadler MR, Haines JE, Eisen MB (2017) Convergence of topological domain boundaries, insulators, and polytene interbands revealed by high-resolution mapping of chromatin contacts in the early *Drosophila melanogaster* embryo. Elife 6. 10.7554/eLife.29550 10.7554/eLife.29550 PMC5739541 29148971

[CR75] Su QP, Zhao ZW, Meng L (2020). Superresolution imaging reveals spatiotemporal propagation of human replication foci mediated by CTCF-organized chromatin structures. PNAS.

[CR76] Su TT, O’Farrell PH (1998). Chromosome association of minichromosome maintenance proteins in *Drosophila* endoreplication cycles. J Cell Biol.

[CR77] Tao L, Dong Z, Leffak M et al (2000) Major DNA replication initiation sites in the c-myc locus in human cells. J Cell Biochem 78:442–457. 10.1002/1097-4644(20000901)78:3<442::aid-jcb9>3.0.co;2-1 10.1002/1097-4644(20000901)78:3<442::aid-jcb9>3.0.co;2-1 10861842

[CR78] Teixeira LK, Reed SI (2017). Cyclin E deregulation and genomic instability. Adv Exp Med Biol.

[CR79] Ulianov SV, Khrameeva EE, Gavrilov AA (2016). Active chromatin and transcription play a key role in chromosome partitioning into topologically associating domains. Genome Res.

[CR80] Vatolina TY, Boldyreva LV, Demakova OV et al (2011) Identical functional organization of nonpolytene and polytene chromosomes in *Drosophila melanogaster*. PLoS One 6 10.1371/journal.pone.0025960 PMC3191165 22022482

[CR81] Wang W, Klein K, Proesmans K (2021). Genome-wide mapping of human DNA replication by optical replication mapping supports a stochastic model of eukaryotic replication. Mol Cell.

[CR82] Weinreich M, Palacios DeBeer MA, Fox CA (2004). The activities of eukaryotic replication origins in chromatin. Biochim Biophys Acta.

[CR83] Weisshart K, Fuchs J, Schubert V (2016). Structured illumination microscopy (SIM) and photoactivated localization microscopy (PALM) to analyze the abundance and distribution of RNA polymerase II molecules on flow-sorted Arabidopsis nuclei. Bio-protocol.

[CR84] Wu P-YJ, Nurse P (2009). Establishing the program of origin firing during S phase in fission yeast. Cell.

[CR85] Zhimulev IF (1999). Genetic organization of polytene chromosomes. Adv Genet.

[CR86] Zhimulev IF, Belyaeva ES, Makunin IV et al (2003a) Influence of the *SuUR* gene on intercalary heterochromatin in *Drosophila melanogaster* polytene chromosomes. Chromosoma 111:377–398. 10.1007/s00412-002-0218-0 10.1007/s00412-002-0218-0 12644953

[CR87] Zhimulev IF, Belyaeva ES, Makunin IV et al (2003b) Intercalary heterochromatin in *Drosophila melanogaster* polytene chromosomes and the problem of genetic silencing. Genetica 117:259–270. 10.1023/a:1022912716376 10.1023/a:1022912716376 12723705

[CR88] Zhimulev IF, Vlassova IE, Belyaeva ES (1982) Cytogenetic analysis of the 2B3-4--2B11 region of the X chromosome of *Drosophila melanogaster*. III. Puffing disturbance in salivary gland chromosomes of homozygotes for mutation l(1)pp1t10. Chromosoma 85:659–672. 10.1007/BF00330779 10.1007/BF00330779 6813059

[CR89] Zhimulev IF, Zykova TY, Goncharov FP et al (2014) Genetic organization of interphase chromosome bands and interbands in *Drosophila melanogaster*. PLoS ONE 9 10.1371/journal.pone.0101631 PMC4114487 25072930

[CR90] Zykova TY, Levitsky VG, Belyaeva ES, Zhimulev IF (2018). Polytene chromosomes—a portrait of functional organization of the *Drosophila* genome. Curr Genomics.

