# Supplementary material for: Super-resolution microscopy reveals stochastic initiation of replication in Drosophila polytene chromosomes
Source: Chromosome Res. 2022 Feb 28;30(4):361–83. doi: 10.1007/s10577-021-09679-w (PMC9771856; doi:10.1007/s10577-021-09679-w)
Supplement: Supplementary file 1 — (DOCX 4.15 MB) [file 10577_2021_9679_MOESM1_ESM.docx]

**10577_2021_9679_MOESM1**

**Supporting information guide**

**10577_2021_9679_MOESM2 Supplementary Movie 1**: Early replication in wild-type chromosome 2L.

**10577_2021_9679_MOESM3** **Supplementary Movie 2:** Early replication in the distal part of chromosome 2L shown in Figure 5.

**10577_2021_9679_MOESM4** **Supplementary** **Movie 3:** Enlarged INT 21C1-2/21D1-2 presented in Figure 5.

**10577_2021_9679_MOESM5** **Supplementary** **Movie 4:** Enlarged INT 21D1-2/21E1-2 depicted in Figure 5.

**10577_2021_9679_MOESM6** **Supplementary Movie 5:** Enlarged INT 21E1-2/22A1-2 displayed in Figure 5.

**10577_2021_9679_MOESM7** **Supplementary Fig**ures (High quality images are available as separate files).

**10577_2021_9679_MOESM8**

**
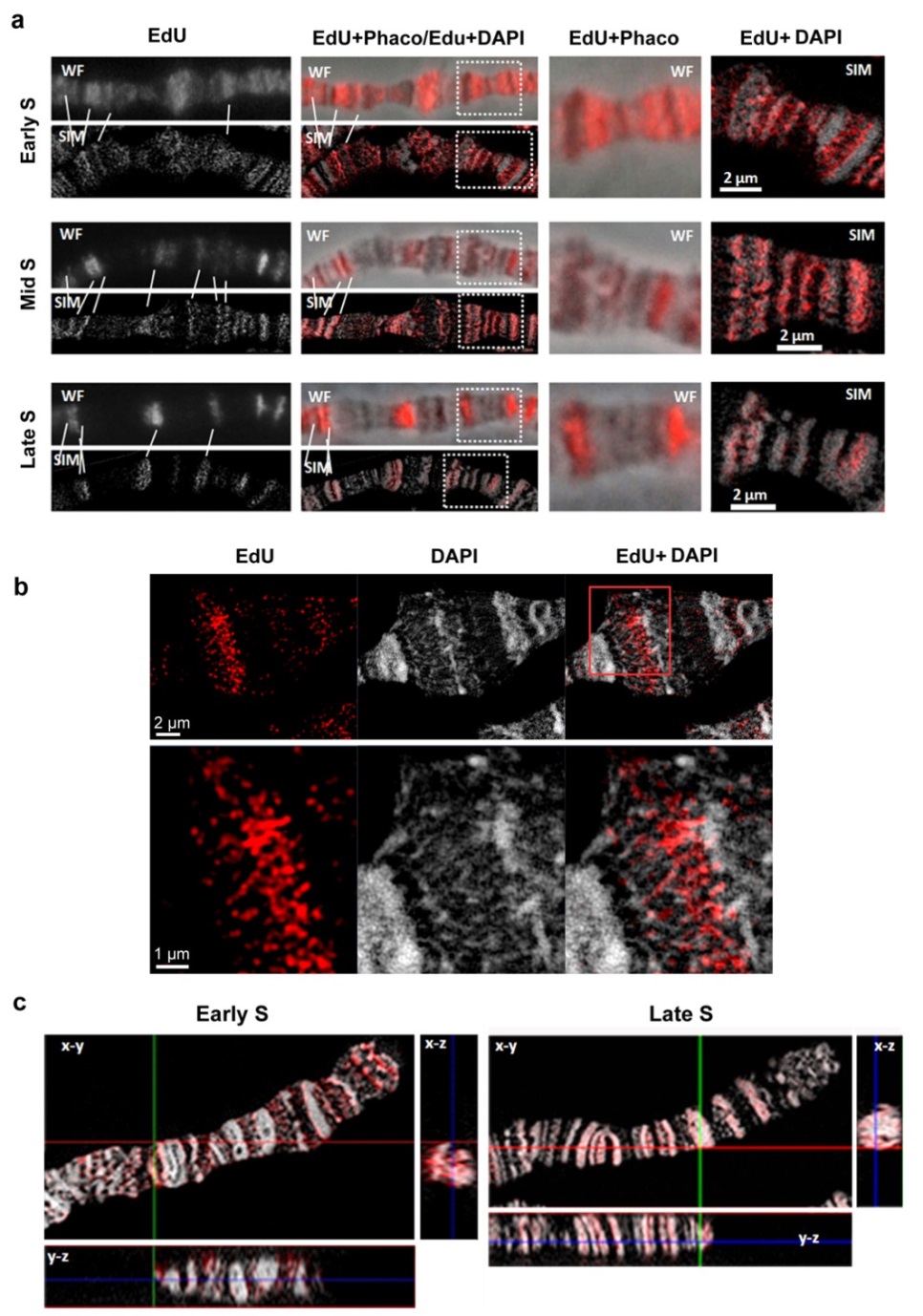
**

**Supplementary Fig. 1** **3D-SIM allows detecting more structural details in comparison with wide-field WF microscopy. (a)** Comparison of early, middle, and late S-phase replication patterns of region 46A-48A (chromosome 2R of wild-type larvae) imaged by WF microscopy and 3D-SIM. The increased resolution achieved by 3D-SIM is especially notable in the enlarged regions (rectangles) on the right. The fluorescence EdU, WF, and SIM images were merged with phase contrast (Phaco) and fluorescence DAPI images, respectively. **(b)** The enlarged region showing very early replication demonstrates the significantly increased resolution achieved by 3D-SIM compared to WF microscopy. Hsp70-CycE; SuUR larvae, 90 min after heat shock. **(c)** Ortho-view of the distal region of wild-type chromosome 2L in early and late S-phase stages. In each chromosome, two different ortho-view positions are visualized. Note that the chromosomes keep their 3D shape as indicated in the x-z and y-z cross-sections. EdU is present throughout the entire chromosome volume.

**10577_2021_9679_MOESM9**

**
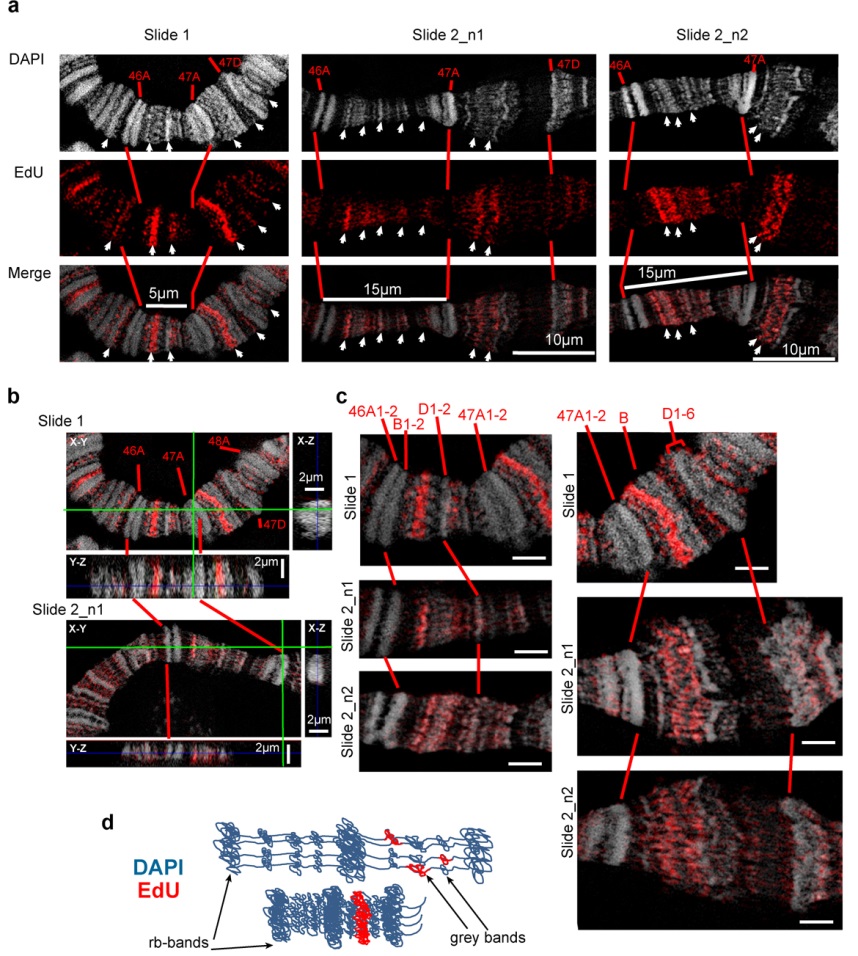
**

**Supplementary Fig. 2 The effect of chromosome stretching on the replication signal shape in *Hsp70-CycE; SuUR* larvae, 90 min after heat shock**.

**(a)** A segment of chromosome 2R including regions 46A-47D, from two squash preparations (Slides 1 and 2). On Slide 1, region 46A-47A is stretched threefold less than in the two examples from Slide 2. This means that no strong chromatin fiber displacement happens along the chromosome axis. Via the chromosome stretching, polytene chromosome band/interband structure becomes more obvious, and the EdU signals spread more within the thin bands. **(b)** Stronger chromosome stretching induces flattening, as evidenced by the ortho-view of the chromosomes in (**a)**. **(c)** At higher magnification, a comparison of two selected chromosome regions from the same three nuclei as in **(a)** reveals a difference in the shape of the signals between the less stretched (top) and more stretched (two lower) chromosomes. **(d)** The scheme explaining the impact of chromosome stretching on the apparent local replication pattern. Stretching visualizes compact and less compact regions along the chromosome axis. EdU labeling becomes visible at different grey band positions in a chromatid, with the formation of a clear-cut band/interband structure. In less stretched chromosomes, the spatial interactions between chromosome regions within the INT are preserved. Replication sites from different chromatids stay together and form replication factories. The band/interband pattern is not discernible on the less stretched chromosomes

**10577_2021_9679_MOESM10**


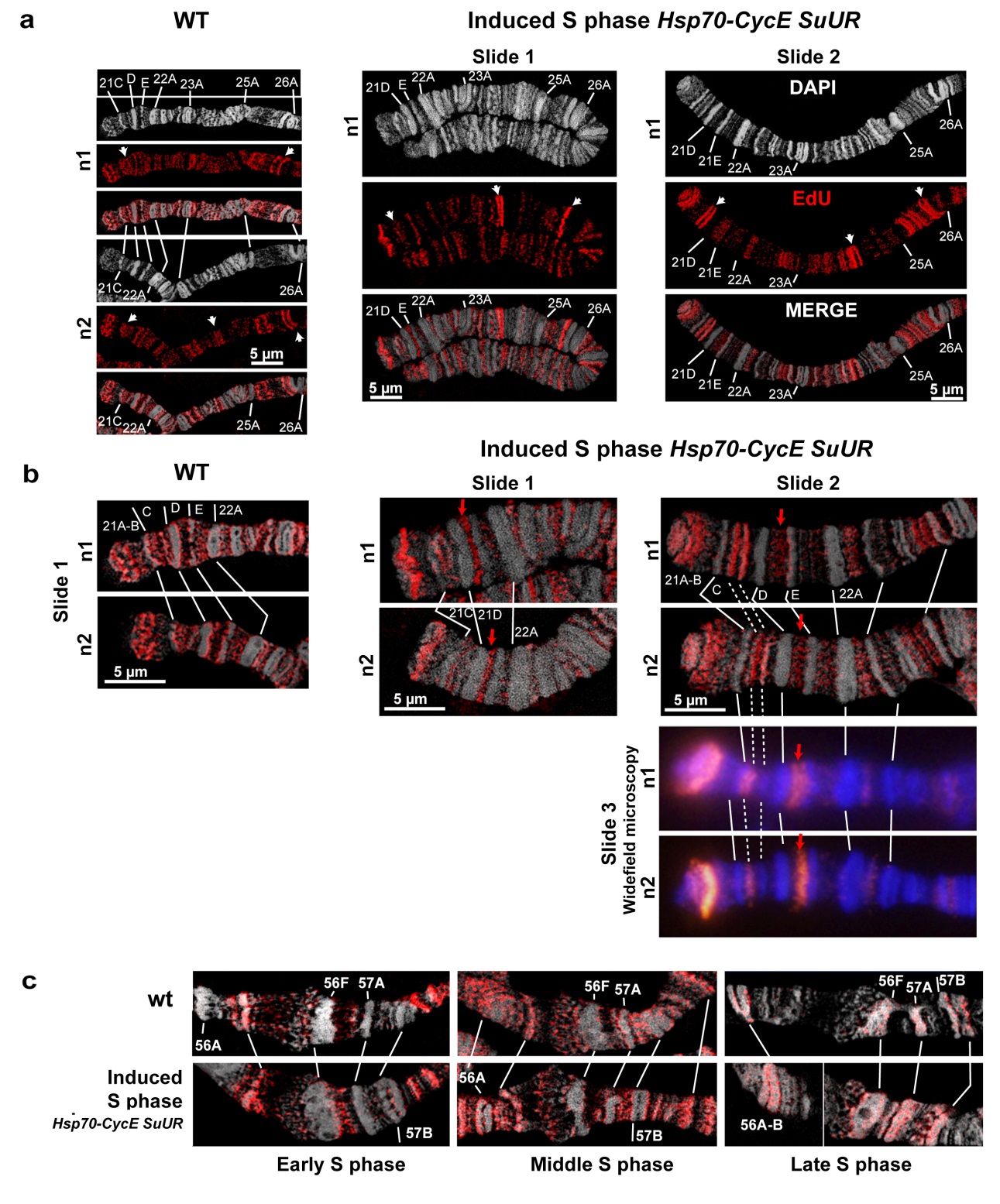


**Supplementary Fig. 3** Replication patterns in wild-type (WT) larvae without heat shock and in *Hsp70-CycE; SuUR* larvae after S-phase induction by heat shock.

**(a)** In contrast to WT, *Hsp70-CycE; SuUR* larvae with the heat shock–induced S phase (90 min after heat shock) feature an additional round of polytenization leading to larger and betterstructured polytene chromosomes. **(b)** The early replication patterns are similar on the same slide but may vary among different slides as exemplified in the distal part of chromosome 2L. The red arrow points to a varied signal in INT 21D1-2/21E1-2.

**(c)** The replication patterns are similar between nuclei from WT larvae and *Hsp70-Cyc; E SuUR* larvae 60 to 180 min after heat shock (induced (IND) S phases), as indicated by the patterns of early, middle, and late S phases of region 56A-57B on chromosome 2R. Late replication patterns were noted in cells that were already in the S phase before heat shock

**10577_2021_9679_MOESM11**


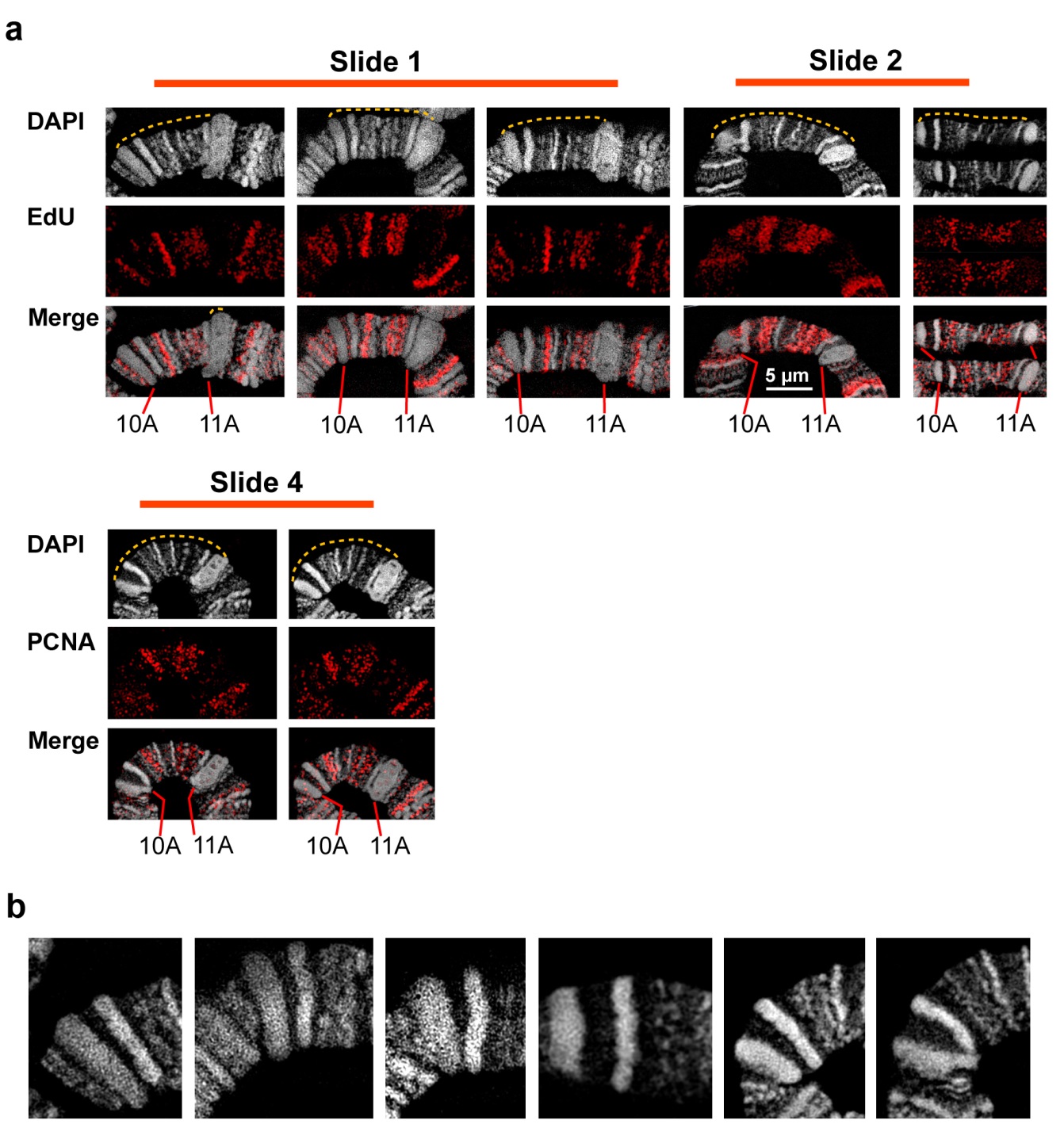


**Supplementary Fig. 4** Reproducibility of the polytene chromosome morphology and early replication patterns in the 10A-11A region of chromosome X as detected by the EdU incorporation assay and PCNA immunolabeling. (**a**) The seven photographs originate from three preparations (Slides 1, 2, and 4; the slide numbering is identical to that in Supplementary Figures 2 and 3). *Hsp70-CycE SuUR* larvae, 90 min after heat shock.

On Slide 1, the signals are arranged as bright lines perpendicular to the chromosome axis but as dispersed spots on Slides 2 and 4. On Slide 2, the chromosome is more stretched than on Slide 1. (**b**) Morphology of 10A1-2 and 10B1-2 bands (enlarged fragments of chromosomes stained with DAPI from a)

**10577_2021_9679_MOESM12**

**
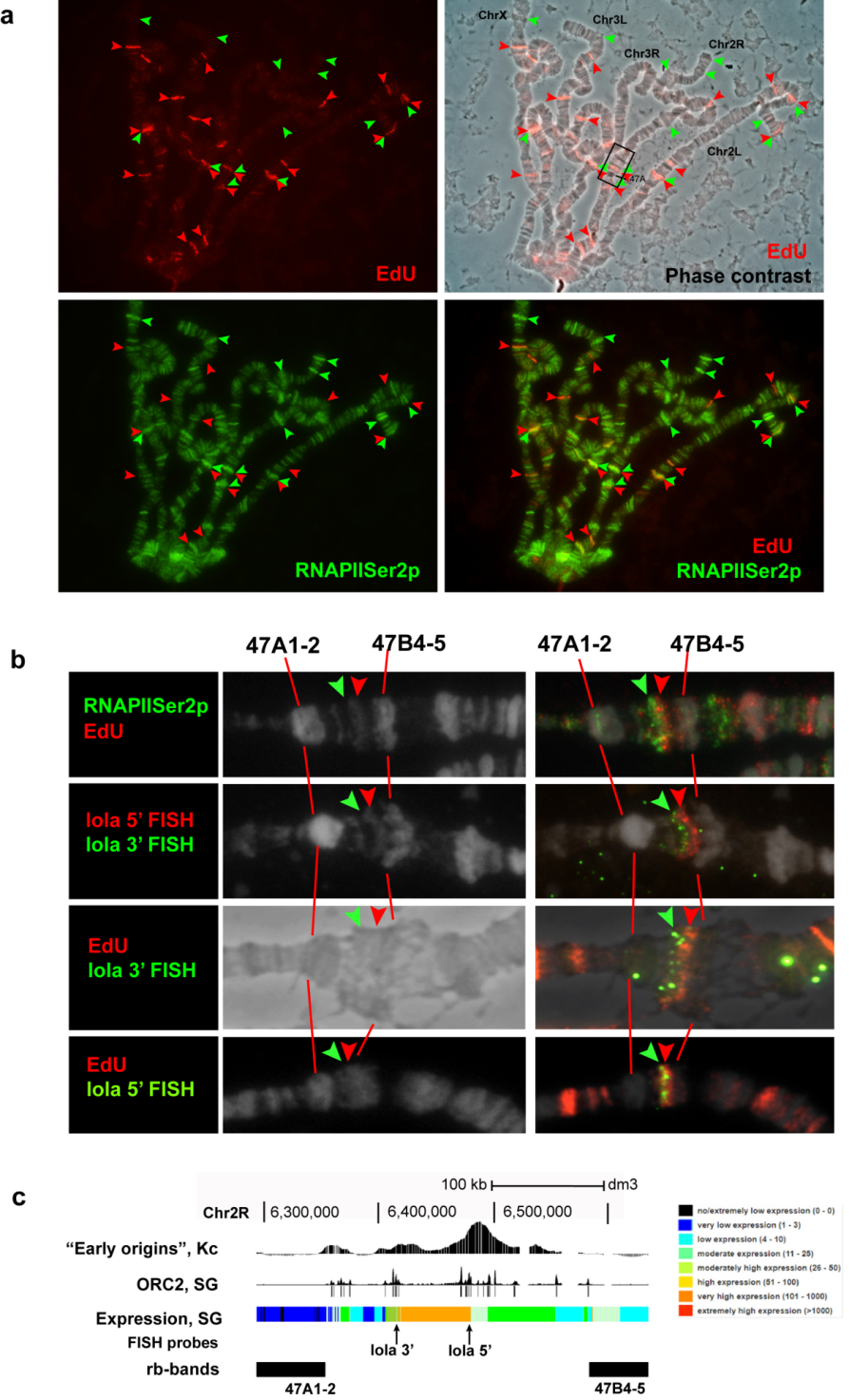
**

**Supplementary Fig. 5** Simultaneous detection of very early replication (EdU) and the active RNA polymerase II variant phosphorylated at serine 2 (RNAPIIser2ph). (**a**) Nucleus at the very early replication stage in *Hsp70-CycE; SuUR* larvae, 90 min after heat shock. Ca. 30 bright EdU sites are present, but significantly more active transcription signals occur. Some of them colocalize, as exemplified for region 47 of chromosome 2R (rectangle in the phase contrast image). (**b**) At locus 47AB efficient early replication initiation starts in the vicinity of the promoter of the actively transcribed *lola* gene. The EdU signal partially coincides with the probe corresponding to the 5’ end of the gene, but lies distant from the probe corresponding to its 3 'end **(c)** Schema of the INT between rb-bands 47A1-2 and 47B4-5. The genomic coordinates of the rb-bands (Kolesnikova et al. 2018), ORC2 binding sites (Sher et al., 2012), “Early origins in Kc” (MacAlpine et al., 2010) and of the lola 3’ and lola 5’ FISH probes are indicated. Data on gene expression in salivary glands (SG) of third instar larvae originate from the ModEncode project.

**10577_2021_9679_MOESM13**


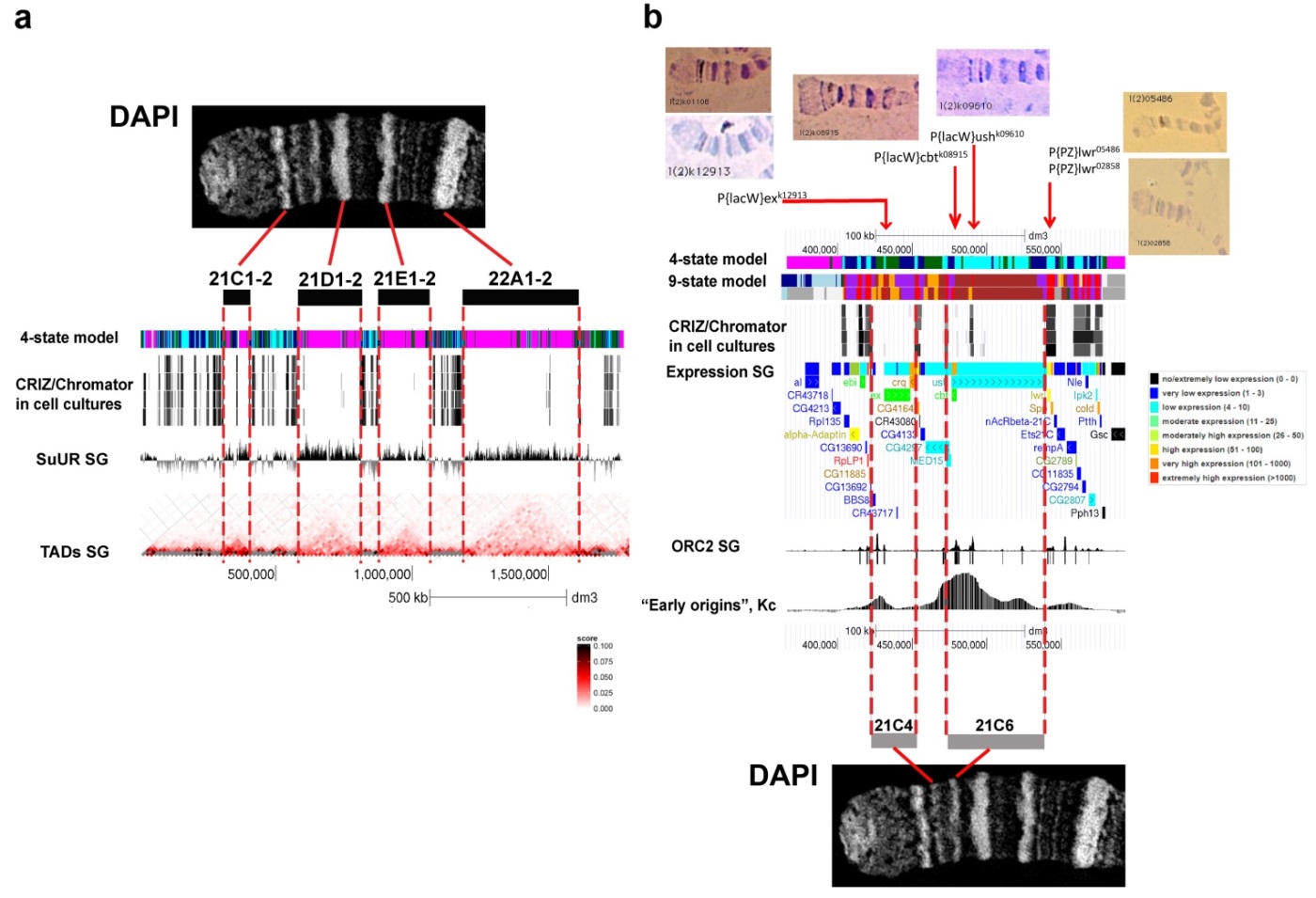


**Supplementary Fig. 6** Localization of polytene chromosome bands in the distal part of chromosome 2L on the *D. melanogaster* genome map.

**(a)** Mapping of rb-bands by an algorithm developed by Kolesnikova et al. (2018). The optical section of a 3D-SIM image stack depicts a fragment of chromosome 2L stained with DAPI. Four very compact bands, 21C1-2, D1-2, E1-2, and 22A1-2, are marked. Below, on the scale of the genomic map, the predicted genomic coordinates of these bands are given, as is a distribution of chromatin properties in the respective region, enabling one to determine the coordinates. The 4-chromatin-states model postulates that all four bands are enriched with ruby chromatin and can be assigned to rb-bands. The presence of the CHRIZ protein in four cell cultures (data from the ModEncode project) is an important marker of polytene chromosome interbands and accordingly is a marker of the band boundaries. Regions of SuUR protein enrichment in SG cells [data from Maksimov et al. (2014)] are also a good marker of rb-bands and their boundaries. A fragment of the contact matrix constructed according to the results of Hi-C analysis in SG cells (Eagen et al. 2015) suggests that the highlighted regions correspond to well-pronounced TADs. This finding further proves that these regions match rb-bands.

**(b)** Mapping of bands 21C4 and 21C6 in the chromosome 2L fragment. These bands are less compact than the rb-bands highlighted in **(a)**. Above are representative photographs of *in situ* hybridization assays of P inserts from the BDGP project (Spradling et al. 1999) mapped to INT 21C1-2/21D1-2. The coordinates of the inserts are described by Spradling et al. (1999), and the images were kindly provided by Tod Laveltry. These images helped to approximately localize 21C4 and 21C6 bands on the genomic map (vertical red dotted lines) and revealed the absence of ruby chromatin. The distribution of red chromatin (active promoters) according to the model of 9-chromatin states (Kharchenko et al. 2011) and of the CHRIZ protein in four cell cultures allowed us to predict the most probable coordinates of the interband. The distribution of the ORC2 protein in salivary glands (SGs) is based on data from Sher et al. (2012). The distribution of newly synthesized DNA in the presence of hydroxyurea (“Early origins”) in S2 cells was published by MacAlpine et al. (2010). “Expression SG” represents the gene expression level in SGs of third instar larvae. Color coding represents expression levels (data from the ModENCODE project)

**10577_2021_9679_MOESM14**


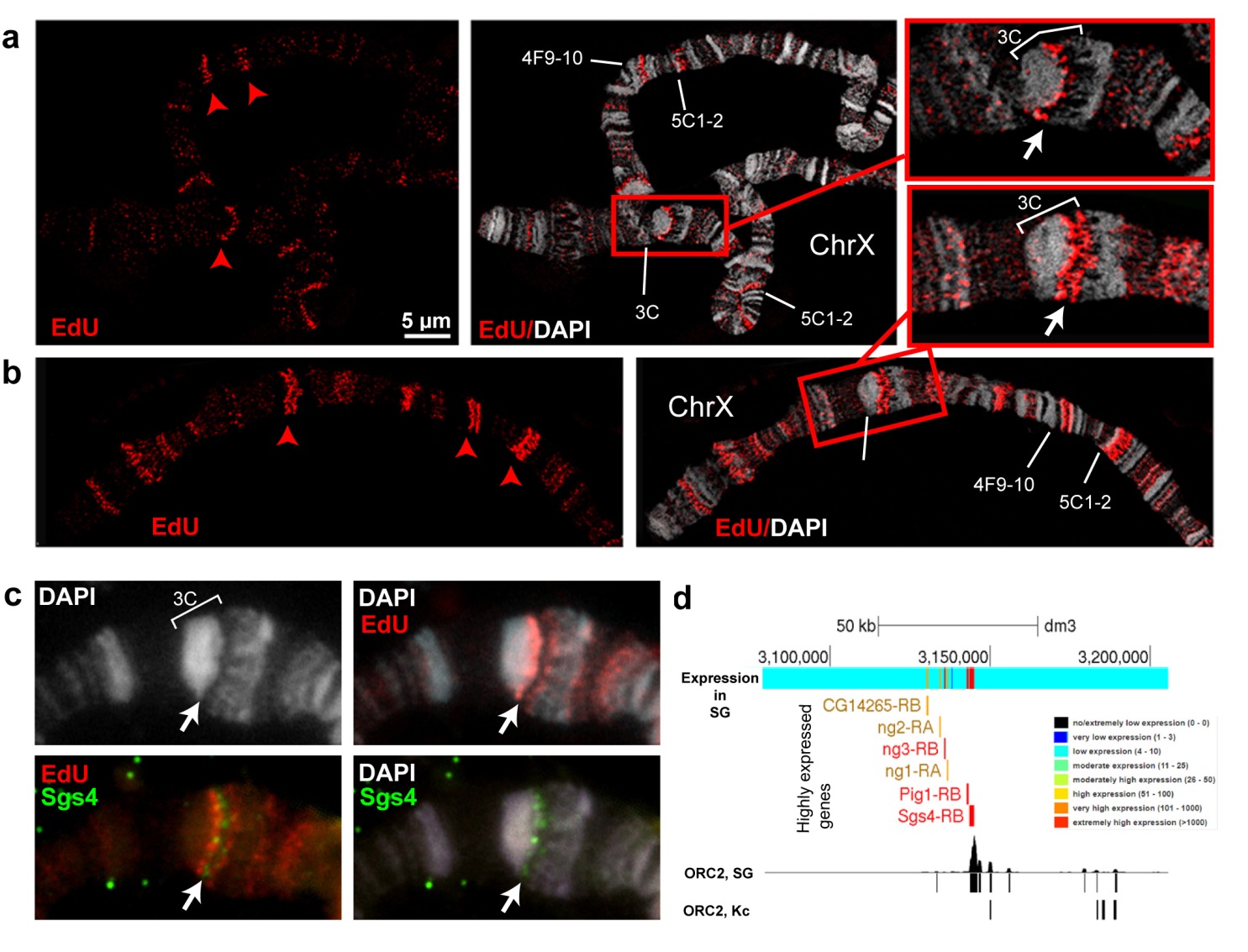


**Supplementary Fig. 7 The *Sgs4* gene region is an example of efficient local replication initiation** (Scenario 1 in figure 5b). **(a)** At very early replication paired signals are not yet visible on a X chromosome fragment, but slightly later they occur **(b)**. The red frames indicate the 3C locus of both X chromosomes. **(c)** Simultaneous detection of early replication (stage of paired EdU signals) and the *Sgs4* gene via FISH at locus 3C. **(d)** Part of the genomic map of the *Sgs4* gene region containing a cluster of genes actively expressed in third larval instar salivary glands. A cluster of ORC2 sites detected in salivary glands is not typical for the Kc cell line. The coincidence of the highest ORC2 peak with the *Sgs4* gene is obvious.

**10577_2021_9679_MOESM15**


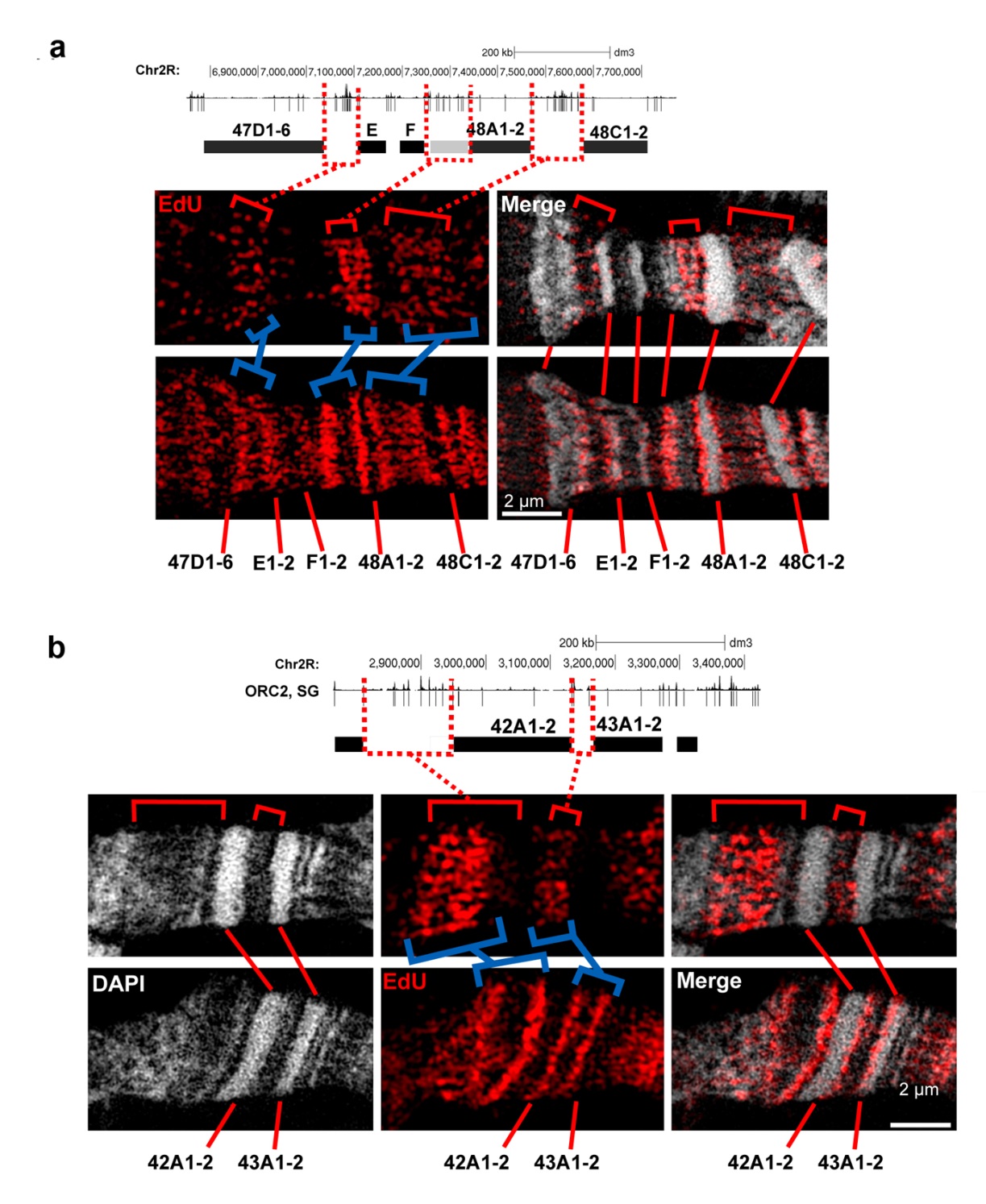


**Supplementary Fig. 8. Replication dynamics at the earliest S-phase stages within INTs 90 min after heat shock.** **(a)** Between rb-bands 47D1-6 and 48C1-2, at an earlier stage (top), three INTs (denoted by red brackets) contain multiple point signals evenly distributed across the INTs. At a later stage (bottom), the same INTs accumulate signals along the edges of the INT, but also many EdU signals are still present throughout the entire INT, as especially noticeable in INT 48A1-2/C1-2. **(b)** A similar dynamics of transitions from diffuse signals to signals accumulated at the INT edges occur in the INTs surrounding rb-band 42A1-2.

**10577_2021_9679_MOESM16**


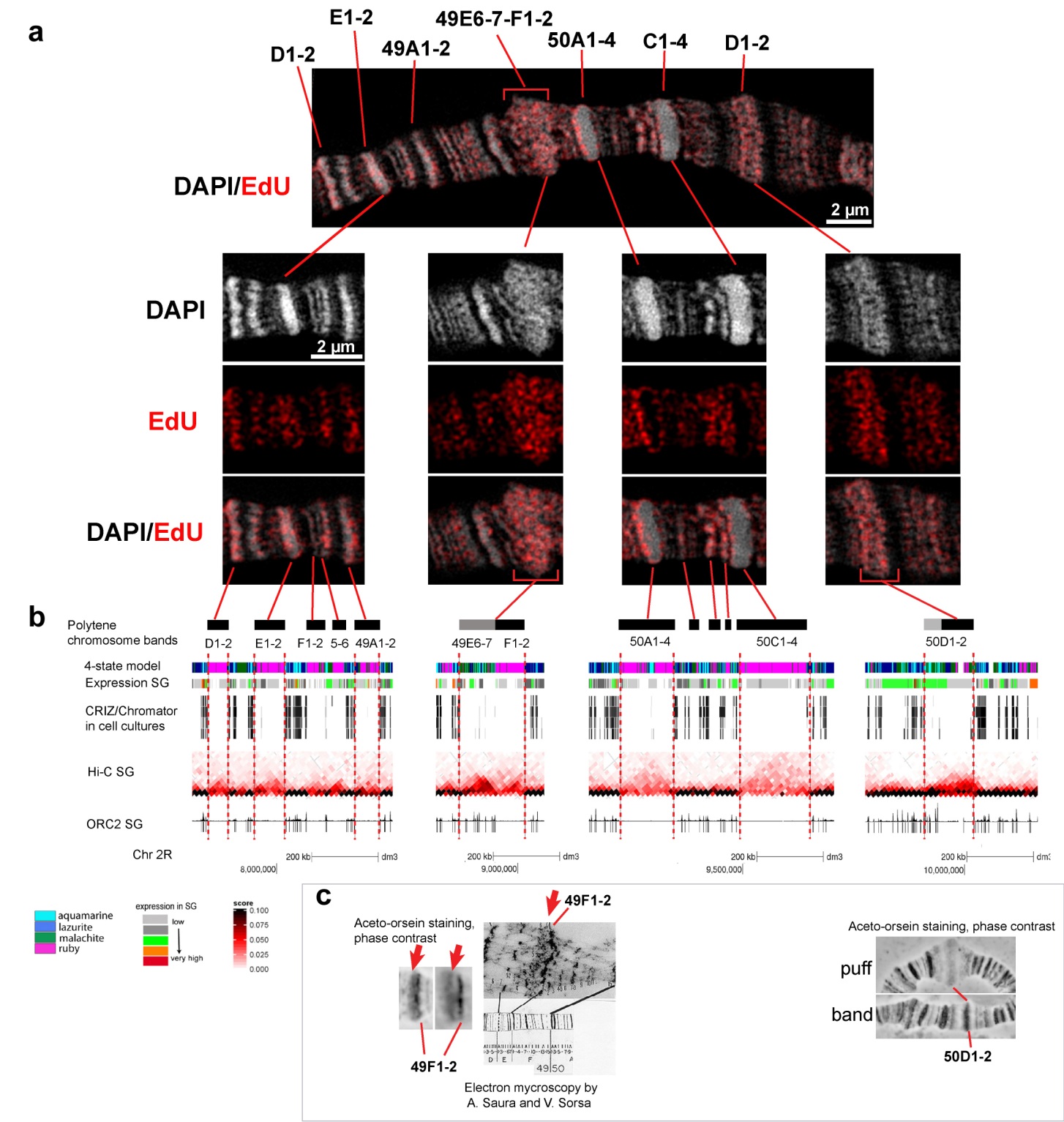


**Supplementary Fig. 9** Only pronounced rb-bands replicate from their periphery to the interior.

**(a)** An optical slice with enlarged regions of a 3D-SIM image stack of region 48D-50D (chromosome 2R) stained by DAPI and EdU incorporation 3 h after heat shock. Within the enlarged regions, rb-bands with this replication pattern can be identified based on genomic coordinates (**b**, **c**).

**(b)** Genomic coordinates of the regions showing compact bands (black rectangles) can be assigned to rb-bands because according to the 4-chromatin-states model (Zhimulev et al. 2014), regions enriched with ruby chromatin (magenta) are evident. Color coding illustrates expression levels in salivary glands (Expression SG) of third instar larvae in the respective regions (data from the ModENCODE project). The presence of the CHRIZ protein noted in four cell cultures (data from the ModEncode project) is an important marker of polytene chromosome interbands and accordingly is an indicator of band boundaries. Fragments of a Hi-C contact matrix originating from SG cells [according to Eagen et al. (2015)] reveal that the highlighted regions correspond to well-pronounced TADs and compact rb-bands. The distribution of ORC2 protein in SGs is based on data from Sher et al. (2012).

**(c)** Characteristic morphology of regions 49F and 50D. In region 49F, the compact band is close to the left-hand loose material; a clear-cut interband cannot be visualized either by aceto-orcein staining or by electron microscopy. Instead, band 50D1-2 may form a puff pointing to a high level of expression

**10577_2021_9679_MOESM17**

**
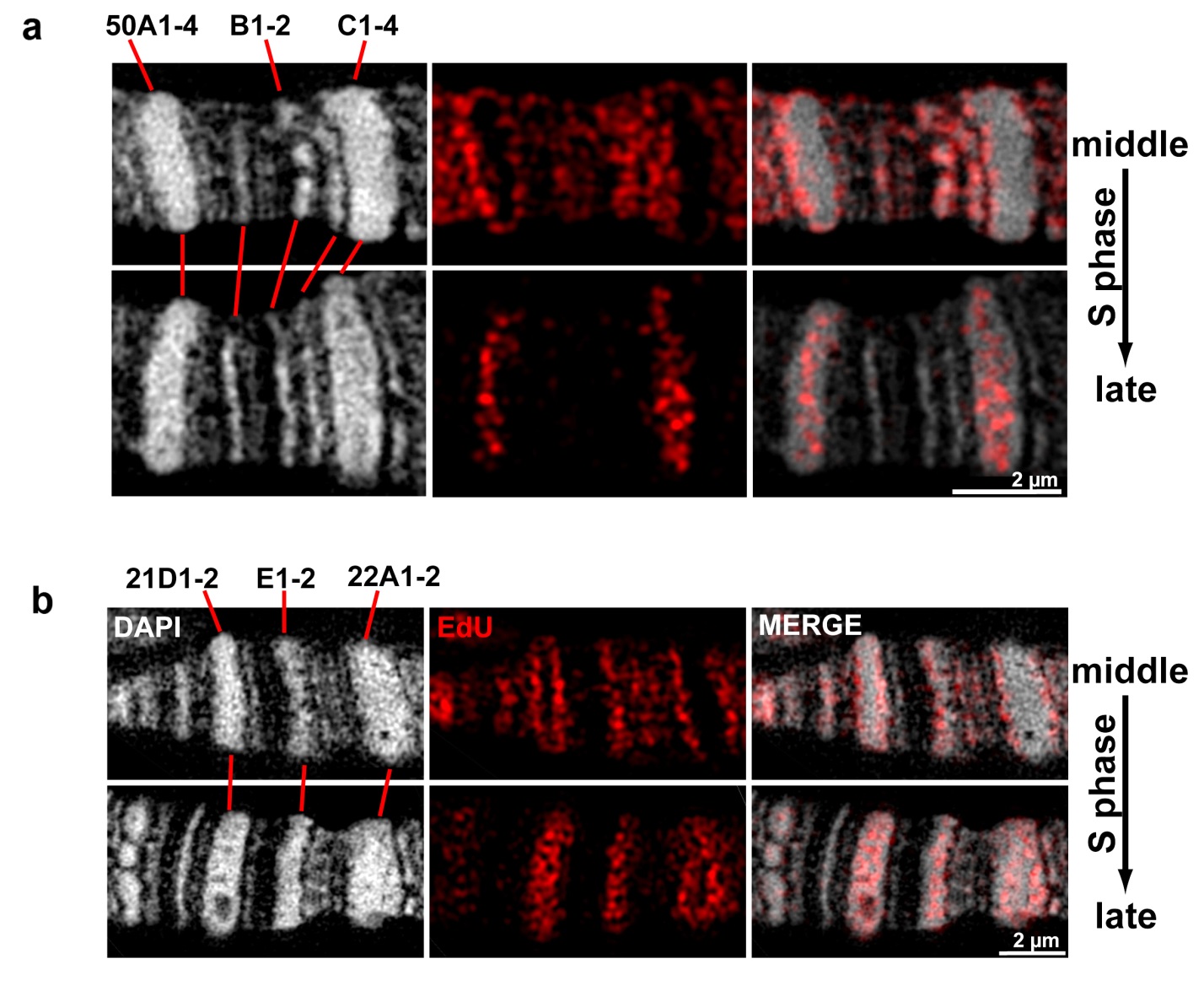
**

**Supplementary Fig. 10 Two examples of mid-late S phase replication dynamics.** **(a)** Region 50A-C. **(b)** Region 21D-22A.

**10577_2021_9679_MOESM18** **Supplementary Text. A replication simulation for a polytene chromosome fragment.** The computer program was written in the Delphi Pascal language
