# Supplementary material for: Super-resolution microscopy reveals stochastic initiation of replication in Drosophila polytene chromosomes
Source: Chromosome Res. 2022 Feb 28;30(4):361–83. doi: 10.1007/s10577-021-09679-w (PMC9771856; doi:10.1007/s10577-021-09679-w)
Supplement: Supplementary file 17 — A replication simulation for a polytene chromosome fragment. The computer program was written in the Delphi Pascal language (DOCX 21.2 kb) [file 10577_2021_9679_MOESM17_ESM.docx]

**Supplementary Text. A replication simulation for a polytene chromosome fragment.** The computer program was written in the Delphi Pascal language

program Computer simulation of replication;

{$APPTYPE CONSOLE}

uses

SysUtils;

const NStrings=1024;

{Graphics parameters}

HScale=10;

VScale=1.2;

Radius=20;

Border = 60; {width of the black boundary}

WhiteSpeed=1;

GraySpeed=(1/2.5)*WhiteSpeed;

BlackSpeed = 0.02*WhiteSpeed;

MiddleSpeed = 0.1*WhiteSpeed;

HStep=16; {time intervals in a minute }

ActiveRatio=Hstep*1024; // variable parameter: how many origins activate in a minute

NGrayStrips = 5;

WidthGrayStrips = WhiteSpeed*Hstep;

RBorder=(2*NGrayStrips+1)* WidthGrayStrips;

Width=RBorder+Border; {-Width <--> Width} type TypeArr = array[1..NStrings] of real;

var

t,n: word;

s: String;

a_left, a_right: TypeArr;

filename: text;

NActive: integer;

function ifOnBlack(x: real): boolean;

begin

ifOnBlack:=(x<=0)or(x>=RBorder);

end;

function ifOnMiddle(x: real): boolean;

begin

//ifOnMiddle := false;

// ifOnMiddle := ( (x/WidthGrayStrips)>5)and( (x/WidthGrayStrips)<6 )or( (x/WidthGrayStrips)>15)and( (x/WidthGrayStrips)<16 );

ifOnMiddle := ( (x/WidthGrayStrips)>5)and( (x/WidthGrayStrips)<6 );

end;

function ifOnGray(x: real): boolean;

var k: integer;

begin

ifOnGray:=((trunc(x/WidthGrayStrips) mod 2)=1);

end;

procedure Initiale(var a: TypeArr);

var i: integer;

begin

for i:=1 to NStrings do a[i]:=0;

end;

procedure Activate(Rat: integer; var al, ar: TypeArr);

var i,j: integer; r: real;

begin

randomize;

i:=0;

while (i<Rat)and (NActive<NStrings) do

begin

j:=random(NStrings)+1;

r:=random(RBorder)+0.5;

if (al[j]=0)and(ar[j]=0)and (not ifOnGray(r)) then

begin

i:=i+1;

al[j]:=r-0.01;

ar[j]:=r+0.01;

NActive:=NActive+1;

end;

end;

end;

procedure Make_a_Step(var al, ar: TypeArr);

var i: integer;

begin

for i:=1 to NStrings do

if (al[i]<>0)or(ar[i]<>0) then

begin

if ifOnBlack(ar[i]) then ar[i]:=ar[i]+BlackSpeed

else

if ifOnMiddle(ar[i]) then ar[i]:=ar[i]+MiddleSpeed

else

if ifOnGray(ar[i]) then ar[i]:=ar[i]+GraySpeed

else

ar[i]:=ar[i]+WhiteSpeed;

if ifOnBlack(al[i]) then al[i]:=al[i]-BlackSpeed

else

if ifOnMiddle(al[i]) then al[i]:=al[i]-MiddleSpeed

else

if ifOnGray(al[i]) then al[i]:=al[i]-GraySpeed

else

al[i]:=al[i]-WhiteSpeed;

end;

end;

procedure Make_one_Step(var al, ar: TypeArr);

var i: integer;

begin

Activate(ActiveRatio div Hstep, al, ar);

for i:=1 to NStrings do

if (al[i]<>0)or(ar[i]<>0) then

begin

if ifOnBlack(ar[i]) then ar[i]:=ar[i]+BlackSpeed

else

if ifOnMiddle(ar[i]) then ar[i]:=ar[i]+MiddleSpeed

else

if ifOnGray(ar[i]) then ar[i]:=ar[i]+GraySpeed

else

ar[i]:=ar[i]+WhiteSpeed;

if ifOnBlack(al[i]) then al[i]:=al[i]-BlackSpeed

else

if ifOnMiddle(al[i]) then al[i]:=al[i]-MiddleSpeed

else

if ifOnGray(al[i]) then al[i]:=al[i]-GraySpeed

else

al[i]:=al[i]-WhiteSpeed;

end;

end;

procedure Minute_Step(var al, ar: TypeArr); // 1-minute interval ...

var t: integer;

begin

for t:=1 to Hstep do // ... divided into Hstep parts for smoothness

begin

Make_one_Step(al,ar);

end;

end;

procedure WriteBoth(var al,ar: TypeArr);

var i: integer;

begin

for i:=1 to NStrings do

writeln(al[i], ' ', ar[i]);

end;

procedure CreateTemplate(var filename: text);

var k: integer;

x1,y1,x2,y2: real;

begin

rewrite(filename);

Writeln(filename, '%!PS-Adobe-3.0 EPSF-3.0');

Writeln(filename, '%%BoundingBox: ',-Border*HScale,' ', 0,' ', Width*HScale,' ', NStrings*VScale:3:3);

Writeln(filename, '%%LanguageLevel: 2');

Writeln(filename, '%%Creator: Pavel Kolesnikov');

Writeln(filename, '%%Title: figure1.eps');

Writeln(filename, '%%CreationDate: ');

Writeln(filename, '%%DocumentProcessColors: Black');

Writeln(filename, '%%EndComments');

Writeln(filename, 0,' 0 moveto ', 0,' ', NStrings*VScale,' lineto ',Width*HScale,' ',NStrings*VScale, ' lineto ', Width*HScale,' ', 0,' lineto closepath stroke');

Writeln(filename, -Border*HScale,' ',0,' moveto ',-Border*HScale,' ',NStrings*VScale, ' lineto ',0,' ',NStrings*VScale,' lineto ',0,' ',0,' lineto closepath 0 setgray fill');

Writeln(filename, RBorder*HScale,' ',0,' moveto ',RBorder*HScale,' ',NStrings*VScale, ' lineto ',Width*HScale,' ',NStrings*VScale,' lineto ',Width*HScale,' ',0,' lineto closepath 0 setgray fill');

for k:=0 to NGrayStrips-1 do

begin

x1:=(2*k+1)*WidthGrayStrips*HScale;

y1:=0;

x2:=(2*k+2)*WidthGrayStrips*HScale;

y2:=NStrings*VScale;

if k<>2 then

Writeln(filename, x1:3:3,' ',y1:3:3,' moveto ',x1:3:3,' ',y2:3:3, ' lineto ',x2:3:3,' ',y2:3:3,' lineto ',x2:3:3,' ',y1:3:3,' lineto closepath 0.8 setgray fill')

else

Writeln(filename, x1:3:3,' ',y1:3:3,' moveto ',x1:3:3,' ',y2:3:3, ' lineto ',x2:3:3,' ',y2:3:3,' lineto ',x2:3:3,' ',y1:3:3,' lineto closepath 0.5 setgray fill');

end;

close(filename);

end;

procedure VisualizeArray(var al,ar: TypeArr; var filename: text);

var i: integer;

begin

append(filename);

for i:=1 to Nstrings do

if (al[i]<>0)or(ar[i]<>0) then

begin

writeln(filename, al[i]*HScale:3:3,' ',i*VScale:3:3,' ', Radius, ' 0 360 arc closepath 1 0 0 setrgbcolor fill');

writeln(filename, ar[i]*HScale:3:3,' ',i*VScale:3:3,' ', Radius, ' 0 360 arc closepath 1 0 0 setrgbcolor fill');

end;

writeln(filename, '%%EOF');

close(filename);

end;

begin

NActive:=0;

Initiale(a_left);

Initiale(a_right);

//

s:='fig0.eps';

assign(filename, s);

Activate(ActiveRatio div Hstep,a_left, a_right);

Make_a_Step(a_left, a_right);

CreateTemplate(filename);

VisualizeArray(a_left, a_right, filename);

//

s:='fig1.eps';

assign(filename, s);

for t:=2 to 1*HStep do

Make_one_Step(a_left, a_right);

CreateTemplate(filename);

VisualizeArray(a_left, a_right, filename);

// *****************************

s:='fig5.eps';

assign(filename, s);

for t:=1 to 4*HStep do

begin

Make_one_Step(a_left, a_right);

end;

CreateTemplate(filename);

VisualizeArray(a_left, a_right, filename);

s:='fig20.eps';

assign(filename, s);

for t:=1 to 15*HStep do

begin

Make_one_Step(a_left, a_right);

end;

CreateTemplate(filename);

VisualizeArray(a_left, a_right, filename);

s:='fig30.eps';

assign(filename, s);

for t:=1 to 10*HStep do

begin

Make_one_Step(a_left, a_right);

end;

CreateTemplate(filename);

VisualizeArray(a_left, a_right, filename);

s:='fig60.eps';

assign(filename, s);

for t:=1 to 30*HStep do

begin

Make_one_Step(a_left, a_right);

end;

CreateTemplate(filename);

VisualizeArray(a_left, a_right, filename);

s:='fig120.eps';

assign(filename, s);

for t:=1 to 60*HStep do

begin

Make_one_Step(a_left, a_right);

end;

CreateTemplate(filename);

VisualizeArray(a_left, a_right, filename);

{ s:='fig180.eps';

assign(filename, s);

for t:=1 to 60*HStep do

begin

Make_one_Step(a_left, a_right);

end;

CreateTemplate(filename);

VisualizeArray(a_left, a_right, filename);

} Writeln('...done');

readln;

end.
